# Distinct biogeographic patterns for bacteria and fungi in association with *Bursaphelenchus xylophilus* nematodes and infested pinewood

**DOI:** 10.1128/spectrum.00778-24

**Published:** 2024-08-20

**Authors:** Yuyu Cao, Nan Yang, Jianfeng Gu, Xingyao Zhang, Jianren Ye, Jianping Chen, Hongjie Li

**Affiliations:** 1 State Key Laboratory for Managing Biotic and Chemical Threats to the Quality and Safety of Agro-products, Key Laboratory of Biotechnology in Plant Protection of Ministry of Agriculture and Zhejiang Province, Institute of Plant Virology, Ningbo University, Ningbo, Zhejiang, China; 2 Ningbo Key Laboratory of Port Biological and Food Safety Testing (Technical Centre of Ningbo Customs/Ningbo Inspection and Quarantine Science Technology Academy), No. 8, Huikang Road, Ningbo, Zhejiang 315100, China; 3 Key Laboratory of Forest Protection of National Forestry and Grassland Administration, Ecology and Nature Conservation Institute, Chinese Academy of Forestry, Beijing 100091, China; 4 Co-Innovation Centre for Sustainable Forestry in Southern China, Forestry and Grassland, College of Soil and Water Conservation, Nanjing Forestry University, Nanjing 210037, China; China Agricultural University, Beijing, China

**Keywords:** microbial diversity, pinewood nematodes, core taxa, biogeographical patterns

## Abstract

Pinewood nematodes (PWN, *Bursaphelenchus xylophilus*) are destructive plant parasitic nematodes that cause pine wilt disease (PWD) by attacking the vascular systems of pine trees, resulting in widespread tree mortality. Research has shown that there are connections between nematode-associated microbes and PWD. Yet the variations in microbial communities across different geographic regions are not well-understood. In this study, we examined the bacterial and fungal communities associated with nematodes and infested wood collected from 34 sites across three vegetation zones in China, as well as samples from the United States, using 16S rRNA and internal transcribed spacer (ITS) gene amplicon sequencing. The predominant genera *Pseudomonas* and *Rhodococcus* were found in nematodes, and *Acinetobacter* was present in the wood of PWD-infected pine trees across China. Network analysis revealed that core bacterial taxa belonged to the Pseudomonadota and Actinomycetota phyla for the nematodes, whereas the Pseudomonadota and Bacteroidota phyla were dominant in the infested wood. Identification of enriched key microbial taxa in nematodes and infested wood across vegetation zones indicates distinct biogeographic microbial community structures and key bacterial species. Although the nematode-associated bacterial community showed consistency across geographic distances, the similarity of the PWD pine trees’ bacterial community decreased with distance, suggesting a spatial correlation with environmental variables. Our findings enhance our understanding of the microbiota associated with pinewood nematode (PWN) and offer valuable insights into PWD management.

**IMPORTANCE:**

Our research uncovered specific bacteria and fungi linked to pinewood nematode (PWN) and infested wood in three different vegetation zones in China, as well as samples from the United States. This sheds light on the critical roles of certain microbial groups, such as *Pseudomonas, Acinetobacter*, and *Stenotrophomonas*, in influencing PWN fitness. Understanding these patterns provides valuable insights into the dynamics of PWN-associated microbiomes, offering potential strategies for managing pine wilt disease (PWD). We found significant correlations between geographic distance and similarity in bacterial communities in the infested wood, indicating a spatial influence on wood-associated microbial communities due to limited dispersal and localized environmental conditions. Further investigations of these spatial patterns and driving forces are crucial for understanding the ecological processes that shape microbial communities in complex ecosystems and, ultimately, for mitigating the transmission of PWN in forests.

## INTRODUCTION

*B. xylophilus* (PWNs) are plant parasitic nematodes that cause destructive damage to pine trees (1). Although PWNs are native to North America, they have become a severe problem in East Asia, especially China (2–4). After more than 40 years of invasions, PWNs have been spread over 17 provinces, causing the death of 19 million trees in total. In 2021, PWD was first found across 10°C isotherms of average temperature and expanded to Jilin Province, Northwest China. The success of PWN invasion and expansion largely relies on mutualistic interactions across multiple kingdoms, including its vector insects, pine hosts, and associated bacterial and fungal microbiota (5, 6).

The PWN-associated microbiota has been considered to play essential roles in nematodes and even be considered a cause for PWD symptoms. During colonization, certain *Pseudomonas* taxa can significantly boost the fecundity, reproduction rate, and body size of PWNs, while *Stenotrophomonas maltophilia* improves the mobility of PWNs under oxidative stress (7–10). Some genera like *Pseudomonas*, *Bacillus*, and *Burkholderia* can produce toxins like phenylacetic acid or pyochelin, which are likely engaged in PWD development (11, 12). The PWN-associated bacterial community has been found to vary across different regions worldwide, i.e., *Pseudomonas* and *Pantoea* were dominant in China (13), while *Bacillus* was dominant in Japan (14). In Korea, all these genera were found to be dominant (15). In the United States, *Chryseobacterium* and *Pigmentiphaga* were first discovered in association with PWNs (16). In Portugal, PWNs were found to carry *Burkholderia*, *Cronobacter*, and *Curtobacterium*. Nonetheless, large-scale geographic investigations based on unculture-dependent approaches remain unappreciated (17), and the biogeographic pattern of microbial communities and core microbes closely related to the hypersensitive reaction of PWD is largely unknown.

Microbial biogeographic patterns and their organizations have been demonstrated to greatly impact ecosystem function and biogeochemical processes. Environmental factors such as geographic distance (18), environmental heterogeneity (19), and dispersal mechanisms (20) strongly influence microbial spatial structuring. Understanding microbial co-occurrence patterns at the continental scale is essential for revealing the different community assembly mechanisms and relevant ecological functions (21). Understanding microbial biogeographic patterns for PWN and PWD pine tree wood will help explore the explicit functions of specific microbes.

In this research, we gathered wild PWNs and infested pine trees from 34 different sites. We used high-throughput Illumina sequencing to analyze the microbial communities of the PWNs and host pine trees. We also investigated the variation in microbial communities of nematodes and infested wood by correlating community dissimilarity with spatial distance matrices. Our findings showed a positive correlation between geographic distance and bacterial community dissimilarity in wood samples, but no significant correlation was observed for nematode-associated microbial communities. This study is essential for identifying the geographically specific key microbial taxa of PWNs and infested wood from distinct sites, with potential applications for tracking the origins of PWNs and/or their infested wood.

## RESULTS

### Microbial composition and diversity of nematodes and infested wood

**Fig 1:**
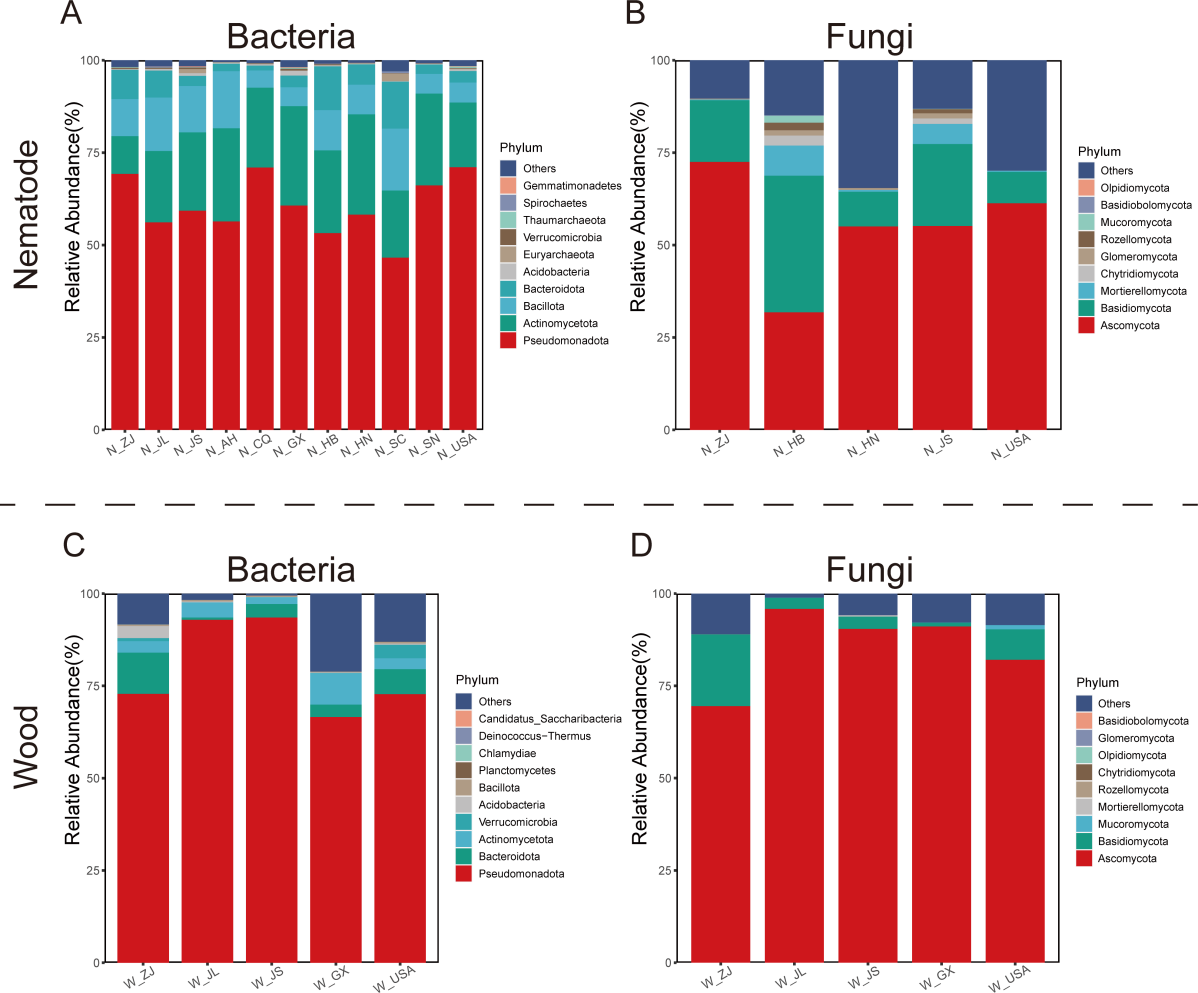
Bacterial and fungal composition at the phylum level of PWNs and host pine trees from diverse regions. (**A**) The top 10 phyla of bacteria and (**B**) the top 10 phyla of fungi of PWNs in relative abundance. (**C**) The top 10 phyla of bacteria and (**D**) the top 10 phyla of fungi of host pine trees in relative abundance. The capital letter N means the nematode, whereas W means the host pine trees. The samples from N_CQ, N_HB, N_AH, N_HN, N_SC, and N_SN were nematode-only.

The bacterial composition of nematodes and infested wood from different locations was similar at the phylum and genus levels, but the proportions varied. The most abundant bacteria in both nematode and infested wood samples at the phylum level was Pseudomonadota. Still, this phylum was more abundant in wood samples than the nematode groups (Fig. 1A and C). The Actinomycetota phylum ranked second in nematodes, with *Pseudomonas* (21.6%) and *Rhodococcus* (11.2%) being major contributors to the distribution patterns of Pseudomonadota and Actinomycetota, respectively (Fig. S1A). *Acinetobacter* (14.4%) was the most abundant genus of the phylum Pseudomonadota in wood (Fig. S1C). Both nematode and infested wood samples showed a high abundance of fungi affiliated with the Ascomycota and Basidiomycota phyla (Fig. 1B and D). However, the dominant fungal taxa varied geographically across regions (Fig. S1B and D).

**Fig 2:**
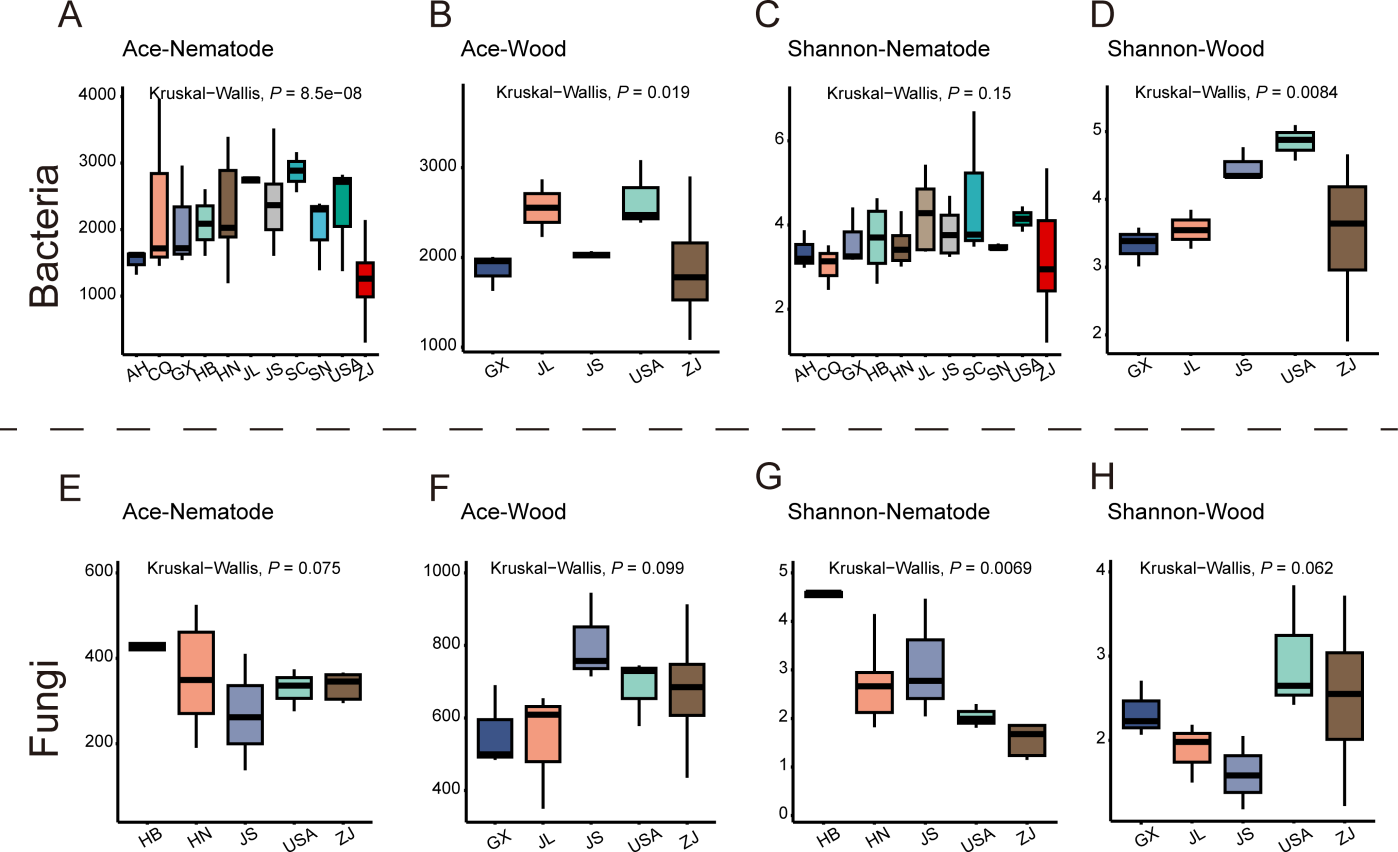
ACE and Shannon indices of bacteria and fungi associated with nematodes and wood from different sites. The Kruskal–Wallis (KW) rank-sum test was used to calculate the varation of ACE and Shannon indices (α  =  0.05).

**Fig 3:**
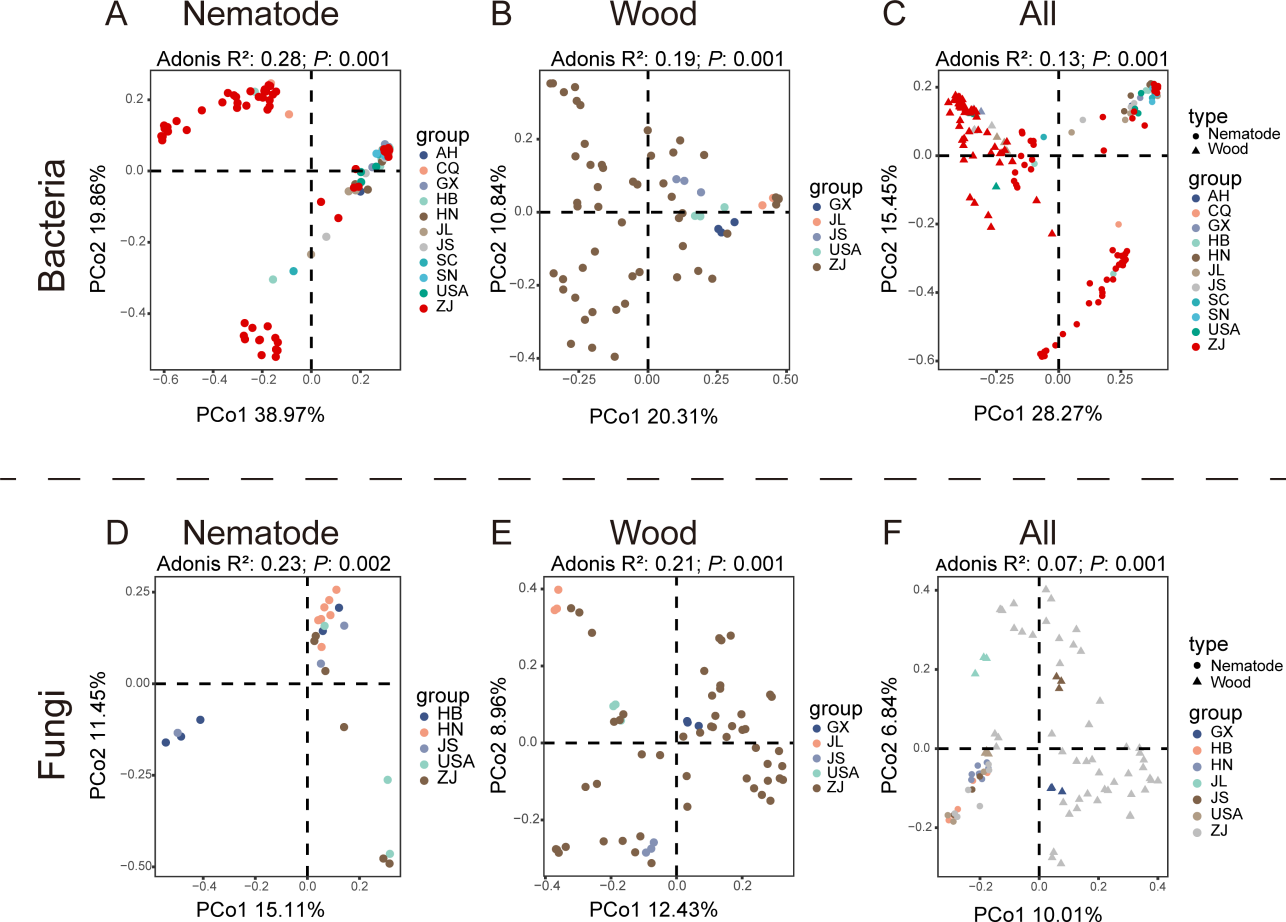
Beta diversity of bacteria and fungi at the ZOTU level of PWNs and host pine trees from various sites. (**A**) PCoA based on Bray–Curtis distances between bacterial samples of PWNs across 11 sites. (**B**) PCoA based on Bray–Curtis distances between bacterial samples of wood across five sites. (**C**) PCoA based on Bray–Curtis distances between bacterial samples of nematode and infested trees. (**D**) PCoA based on Bray–Curtis distances between fungal samples of neamtodes across five sites. (**E**) PCoA based on Bray–Curtis distances between fungal samples of wood across five sites. (**F**) PCoA based on Bray–Curtis distances between fungal samples of nematode and infested trees. All these plots are color-coded according to sites.

Alpha and beta diversity indexes were used to evaluate variations in the community composition among different sampling locations. The ACE index of the bacteria associated with nematode and infested trees varied significantly among different regions (*P* < 0.05), while there was no significant difference in the fungi. The Shannon index of fungi associated with nematodes exhibited significant variation across different regions, whereas no significant differences were observed in the bacteria associated with them. Interestingly, the Shannon index of microbes associated with infested trees exhibited a contrary tendency (Fig. 2). Principal coordinate analysis (PCoA) plots showed significant differences in associated bacterial communities of pinewood nematodes across different sites. Notable differences were also observed in associated fungal communities (Fig. 3A and C). Similar trends were observed in the microbial communities of wood samples (Fig. 3B and E). The dissimilarity of the wood samples vs nematode samples is significant (Fig. 3C and F).

### Geographically core microbial taxa of nematodes and infested wood

**Fig 4:**
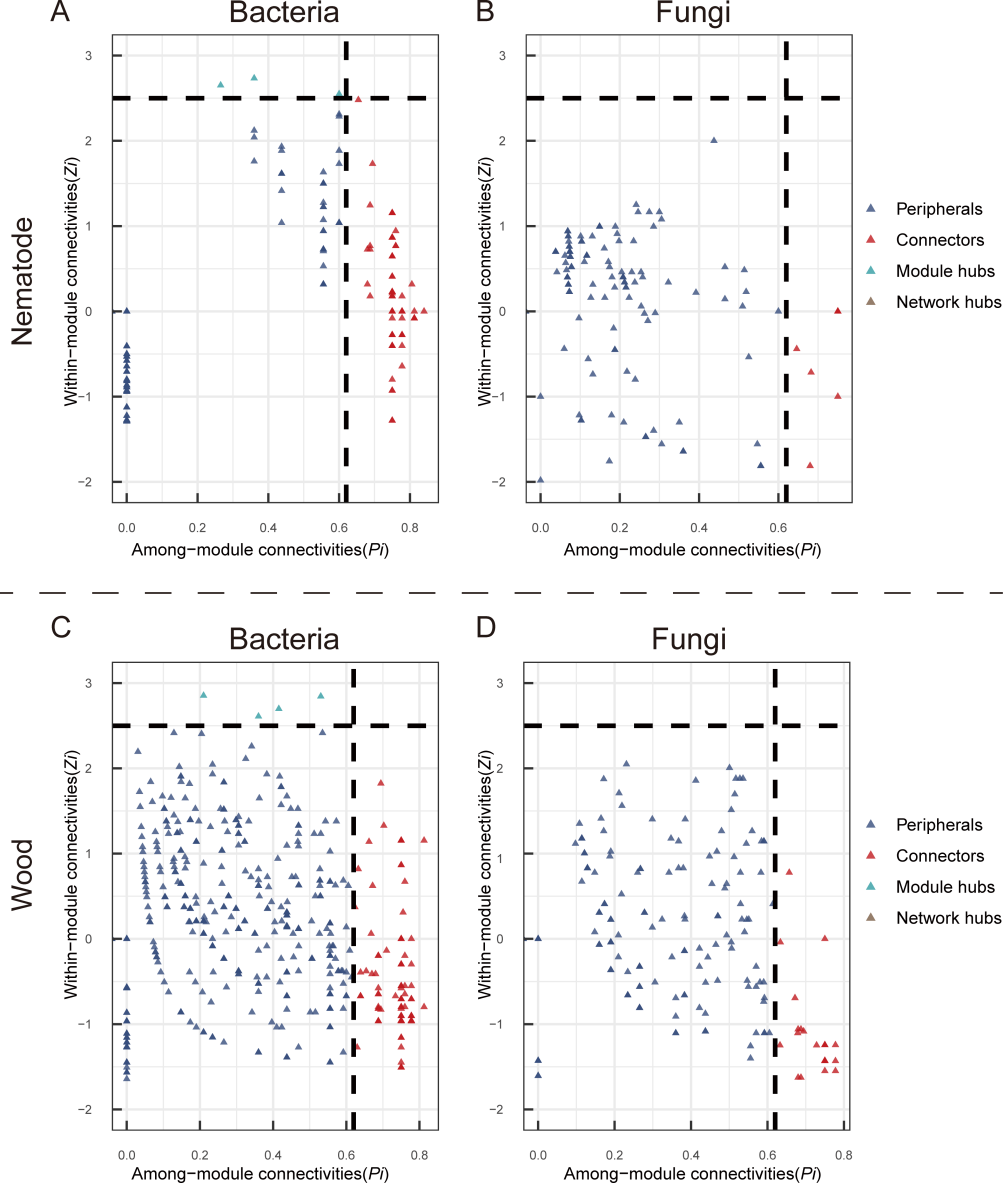
Classification of nodes of interaction networks to identify putative core species within the networks of nematodes (**A and B**) and wood (**C and D**). The analysis was based on the ZOTU level. The dashed lines on the axes indicate the threshold values for determining the core species. Different ZOTUs may have same Zi and Pi. The connectors, module hubs, and network hubs were considered core species. Detailed taxonomic information for module hubs and connectors is listed in Tables S2 and S3.

Core bacterial and fungal taxa (taxa were divided into module hubs, network hubs, and connectors) in nematodes and infested woods were identified by analyzing co-occurrence networks. The dotted lines in axes represented the cutoff thresholds for connectivity of different nodes (Fig. 4). No network hubs were detected in the nematode and wood microbial networks, as shown in Fig. 4. Compared to the fungal interaction network, bacteria formed a more complex co-occurrence network.(Fig. S2). The nematode bacterial network had 89 connectors and 3 module hubs, annotated to 23 genera. More than half (59.8%) of the connectors belonged to the Pseudomonadota phylum (Table S5). Compared to the phylum Pseudomonadota, the phyla Bacillota, Bacteroidota, and Actinomycetota of the core bacterial microbiota are relatively lower, accounting for only 10%, 6.8% and 16% of all the core bacterial microbiota in the nematodes. At the order taxonomic level, the core microbiota of Mycobacteriales (15%) within the phylum Actinomycetota and Clostridiales (4%) within the phylum Bacillota have core microbial quantities only lower than those of Burkholderiales (26.9%) within the phylum Pseudomonadota. Interestingly, Alteromonadales and Ignavibacteriae were identified as core microbiota with the relative abundance of 0.02%. It’s worth noting that only one ZOTU within Ignavibacteriae has been identified as core microbiota. (Fig. 4A; Table S2). The nematode fungal network had only five core nodes, belonging to the *Aureobasidium* genus, and the proportion of this microorganism was 0.92% (Fig. 4B; Table S2). The infested wood bacterial network had 122 core nodes annotated to 22 genera, 68% affiliated with the Pseudomonadota phylum, followed by Bacteroidota (12.2%) and Acidobacteria (5.7%). Unlike the core microbiota of nematodes, the phylum Bacillota was not identified as core microbiota of wood samples. The relative abundance of core microbiota in nematodes is higher than that in infested wood for Moraxellaceae, Erythrobacteraceae, Burkholderiaceae, Sphingomonadaceae, and Weeksellaceae. Conversely, for Pseudomonadaceae, Flavobacteriaceae, and Comamonadaceae, the opposite trend is observed (Table S6). The infested wood fungal network had 23 taxa annotated to 10 genera, with 73.4% belonging to the Ascomycota phylum. (Fig. 4D; Table S3). Additionally, both nematode and wood bacterial networks observed taxa belonging to the same genera, namely, *Acinetobacter*, *Flavobacterium*, *Novosphingobium,* and *Paraburkholderia*.

Using the Linear discriminant analysis Effect Size (LEfSe) method, our analysis has identified 47 bacterial biomarkers for nematodes and 31 bacterial biomarkers for infested wood at the genus level (Fig. S3). Almost half of the bacterial biomarkers (48.9%) found in nematodes belonged to the Pseudomonadota phylum, while the proportion was slightly higher (58%) for infested wood samples (Fig. S3A and B). Additionally, we identified 9 fungal biomarkers for nematodes, most affiliated with Ascomycota (Fig. S3C). In infested wood samples, most fungal biomarkers (63.8%) belonged to the Ascomycota group, while the others were mostly involved in Basidiomycota (Fig. S3D). All the identified biomarkers had LDA scores (log10) greater than 3, with a significance level of *P* < 0.05.

### Geographically key microbial taxa across different vegetation types

**Fig 5:**
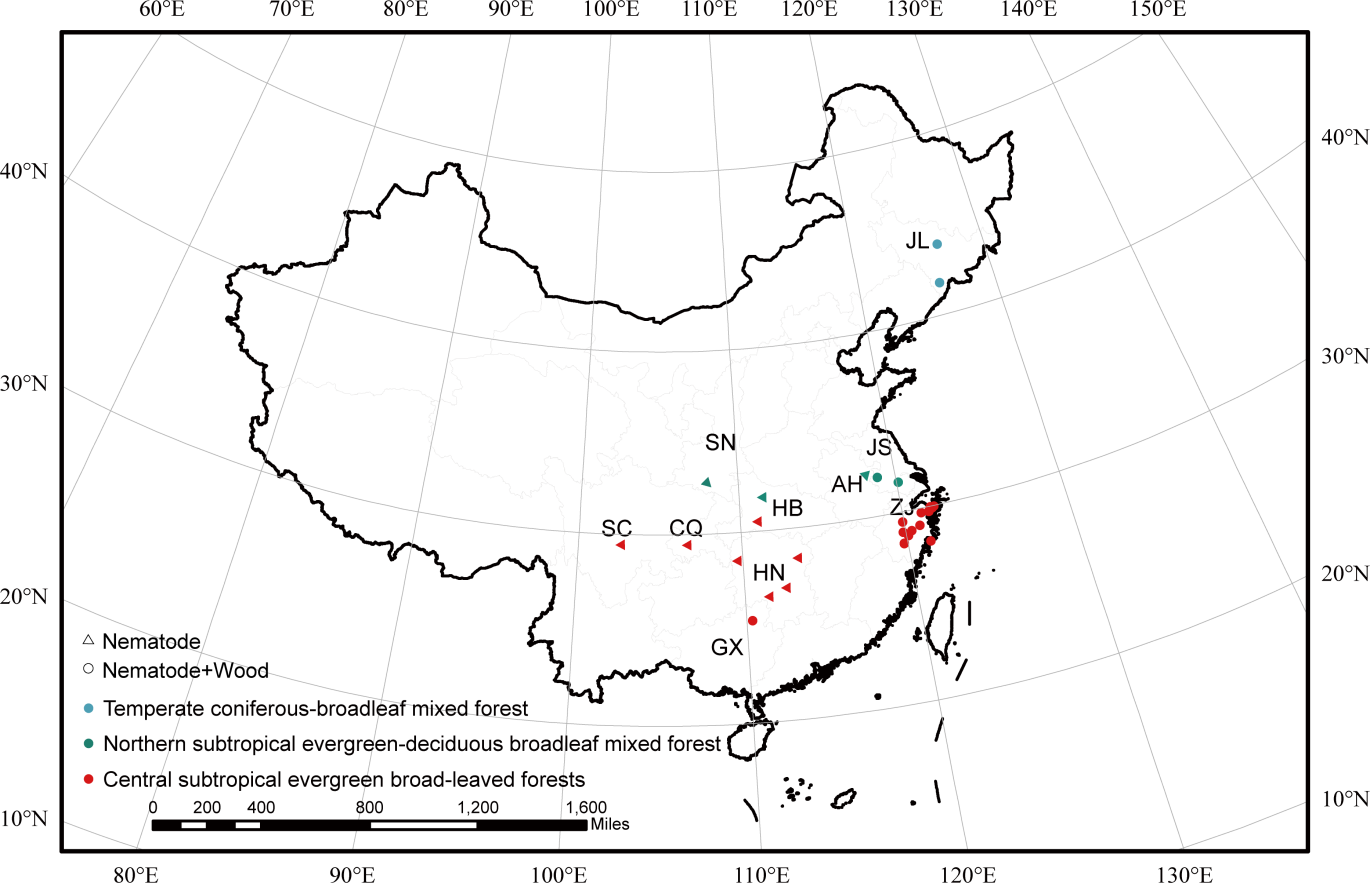
The sampling sites of China were divided into three forest types based on the geographic location. The triangles depicted herein signify the sampling sites containing samples that are exclusively composed of pine wood nematodes, while the circles denoted samples separated from nematodes and wood. The sampling sites were color-coded by vegetation type. This figure was created by using the ArcGIS software (ArcMap 10.8.1) with 1: 1000000 public version basic geographic information data (2021), National Catalogue Service for Geographic Information, National Geomatics Center of China.

We categorized the sampling points in China into three vegetation types, namely, temperate coniferous–broadleaf mixed forest, northern subtropical evergreen–deciduous broadleaf mixed forest, and central subtropical evergreen broad-leaved forest (Fig. 5). Key microbial taxa were identified in pinewood nematodes and infested wood across different vegetation types (Table S4). First, we selected key taxa by analyzing the ZOTUs (zero-radius operational taxonomic units) that appeared in 80% of samples among all samples collected from China. Then, we identified the ZOTUs commonly shared across all sampled sites. In total, 16 bacterial and 1 fungal ZOTUs were commonly shared among the nematodes. For infested wood, 51 bacterial and 1 fungal ZOTUs were commonly shared. We analyzed the enriched ZOTUs based on significant variances across vegetation types, derived from commonly shared ZOTUs. The results indicated that *Ralstonia*, *Pseudomonas*, and *Stenotrophomonas* were enriched in the nematodes of central subtropical evergreen broad-leaved forests and temperate mixed forests. Enrichment of bacteria, including *Acinetobacter*, *Luteibacter*, was observed in the infested wood of these forests. In contrast, no enriched bacterial taxa were observed in nematodes of northern subtropical evergreen–deciduous broadleaf mixed forests, and *Paraburkholderia* was the only enriched taxon in infested wood of this vegetation type (Table S4).

### Geographic patterns of microbial community diversity

We conducted a Mantel test to examine the correlation between community dissimilarity and spatial distance matrices to identify additional spatial sources of variation in the microbial communities of nematodes and infested wood. After examining complete data sets, we noticed that the distance between geographic locations and bacterial community dissimilarity of infested wood had a positive correlation (r = 0.2513, *P* = 0.001). On the other hand, the dissimilarity of microbial communities in nematodes that were collected from the same origin as infested wood did not have any correlation with geographic distance (r = 0.1573, *P* = 0.14) (Fig. 5). Interestingly, the Mantel test revealed that bacterial community dissimilarity of nematodes from all 33 sampling sites across China did not correlate with geographic distance (r = −0.01196, *P* = 0.541) (Fig. S4).

We further performed fitting linear models to test for the effects of latitudinal variables on the richness and Shannon index values (Fig. S5 and S6 ). The bacterial richness indexes of nematodes (R^2^ = 0.0559, *P* = 0.0108) and infested wood (R^2^ = 0.0625, *P* = 0.0302) have been found to be positively correlated with latitude, while fungal richness index values were not significantly affected by latitudinal variables (R^2^ = −0.0088, *P* = 0.3793; R^2^ = −0.0004, *P* = 0.3273). In contrast, concerning the Shannon indexes, only fungal Shannon index values are significantly affected by latitude for nematodes (R^2^ = 0.1912, *P* = 0.0212) and infested wood (R^2^ = 0.0800, *P* = 0.0162).

## DISCUSSION

Our study identified dominant bacterial genera including *Pseudomonas* and *Rhodococcus* for *B. xylophilus* nematodes and *Acinetobacter* for PWD pine tree wood across China. Enriched keystone microbial taxa in nematodes and infested wood across vegetation zones indicate distinct biogeographic microbial community structures. Distance decay relationships revealed unique biogeographic patterns in bacterial and fungal communities. We observed distinct diversity patterns between nematode and wood samples, suggesting that microbial habitats within the same ecosystem can exhibit considerable diversity (22). Differences in nematode-associated microbial communities were notable, but even more pronounced disparities were found in PWD pine trees’ microbial community, suggesting a significant influence of environmental factors on wood microbial diversity, consistent with strong variances of pine tree endophytes.

Evidence for dominant taxa in the bacterial community was observed, such as Pseudomonadota and Actinomycetota for the *B. xylophilus* nematode, with enriched genera in *Pseudomonas* and *Rhodococcus* (Fig. 1 ; Fig. S1). The detection of *Rhodococcus* rather than the closely related taxa *Mycobacterium*, which was earlier reported as enriched in nematodes, suggests likely site-specific differences in *B. xylophilus-*associated microbiomes. *Rhodococcus* in *B. xylophilus* potentially promotes the fitness of nematodes. The observation of the relative abundance of the *Acinetobacter* genus in the infected *Pinus massoniana* wood is consistent with that of previous studies, indicating that several *Acinetobacter* isolates obtained from symptomatic pine trees in Portugal show significant inhibition of plant root growth (23). Compared with prior studies, we observed differences in dominant bacteria; dominant bacteria were distinct among different kinds of host pine trees (24–26); this may be an important factor for the differences in key microbial species within nematode groups (27, 28).

Interestingly, the nematode and infested wood microbial networks lacked network hubs, indicating a decentralized network structure (Fig. 4). Our results, in line with recent observations of the most abundant Burkholderiales rather than early founding of dominant *Pseudomonas*, indicate substantial microbial communities shift along with time. Also, the findings of core taxa with depleted abundance, such as *Alteromonadales* (0.06% in nematode and 0.002% in wood) and Ignavibacteriae (0.02% in nematode and 0% in wood), suggest their potential roles associated with PWN microbiome stability, even though recently identified and remains poorly understood.

Bacteria showed a more complex co-occurrence network than fungi (Fig. S2). This suggests potential cooperation or mutualistic relationships among microbial species, which may enhance the environmental fitness (29).The prevalence of Pseudomonadota in nematode and infested wood samples underscores its potential as a key player in these ecosystems. The fungal biomarkers affiliated with Ascomycota and Basidiomycota suggest the diverse roles different fungal groups play in these environments (30, 31).

Our study clearly shows distinct biogeographic bacterial and fungal taxa patterns associated with nematode and infested wood through the diversity of different communities across vegetation types (Table S4). In central subtropical evergreen broad-leaved forests and temperate mixed forests, nematodes exhibited enrichment with key bacterial taxa, such as *Stenotrophomonas*, *Ralstonia*, and *Pseudomonas*, together with previous findings of similar bacteria consistently isolated from Portuguese *B. xylophilus*, supporting the importance of these nematode-associated bacteria and potential pathogenic roles in the pine wilt disease. Observation of the majorly shared key taxa of *B. xylophilus* among vegetation types, together with the recent report that few microbial taxa filtered by vector beetles’ tracheae, aligns with the longstanding hypothesis that conservative key taxa were associated with nematodes. Nematodes like *C. elegans* show a strong preference for microbiomes that are low colonizers and have been shown to support faster *C. elegans* development time (32). Furthermore, under the high bacterial taxonomic standard, the *C. elegans*’ bacterial community from the same host is similar, regardless of the specific sampling site (33). *C. elegans*’ microbiotas resembled each other even when assembled from different microbial environments (34).

*B. xylophilus* can substantially modify the microbiota structure of its host pine tree, suggesting the complex interactions among parasitic nematodes, host plants, and surrounding environmental variables. These trends highlight microbial communities’ dynamic nature and responsiveness to environmental gradients, such as climate (35), humidity, and soil characteristics (36). Our results revealed that in central subtropical evergreen broad-leaved forests, infested *P. massoniana* wood had key taxa, including *Acinetobacter* and *Luteibacter*. In contrast, the enrichment of *Paraburkholderia* key taxa was observed in northern subtropical evergreen–deciduous broadleaf mixed forests. Taken together, the key taxa patterns of infested wood are well-consistent with corresponding nematodes, implying the essential roles of *B. xylophilus* in regulating the microbiota structures of host pine tree (37).

**Fig 6:**
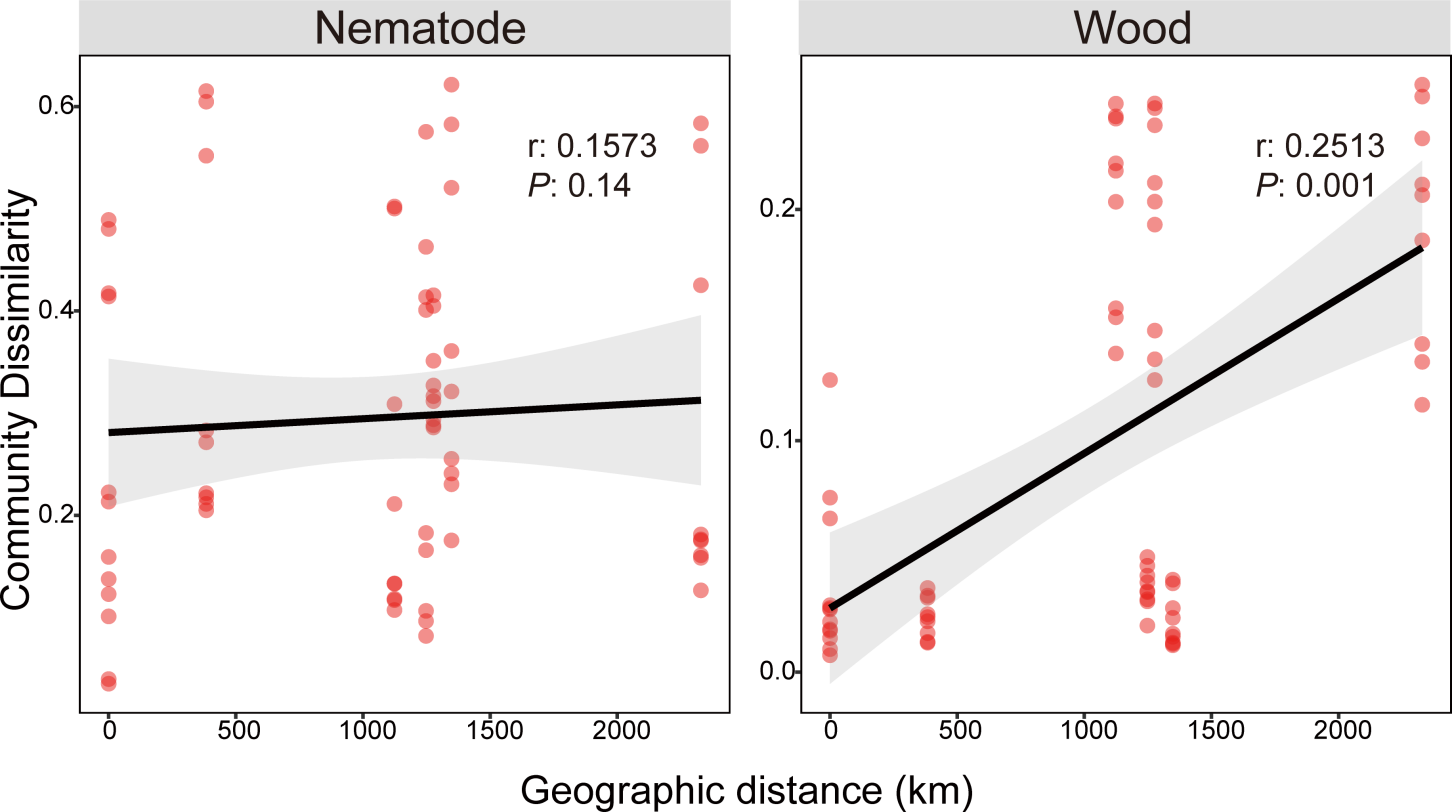
Relationships between the Bray–Curtis dissimilarities of PWNs and wood sharing the same sampling sites with geographic distance. The lines represent the linear regression results. The shaded areas show the 95% confidence interval. The r means the correlation coefficient. When r is greater than 0, it indicates a positive correlation between the geographic distance and community dissimilarity; when it is less than 0, they are negatively correlated.

The results of our study shed light on the spatial dynamics of microbial communities associated with nematodes and wood, revealing intriguing patterns of correlation with geographic distance. The significant correlation between wood communities and geographical distance reflects the influence of geographical distance on the composition and structure of plant communities, such as differences in ecological conditions, climate, and vegetation types between different regions, which may lead to differences in wood communities (Fig. 6). Unexpectedly, we observed no significant correlation between the dissimilarity of microbial communities in nematodes and geographic distance despite sharing the same sample origin as the wood microbial communities. Monroy et al. found that soil nematodes and the soil they inhabit exhibit inconsistent correlations, specifically in community similarity decayed with distance, and that this spatial pattern was not related to changes either in plant community composition or soil chemistry (38). This intriguing discrepancy suggests distinct spatial dynamics between nematode-associated and wood-associated microbial communities, indicating potentially different dispersal mechanisms or environmental drivers shaping their distributions (35). Additionally, our Mantel test results offered further insights, indicating the absence of correlation between bacterial community dissimilarity among nematodes across all 33 sampling locations in China and geographic distance. This could be due to various factors, including the nematodes’ physiology, behavior, and interactions with their environment, which may favor the colonization and proliferation of certain microbial species over others (39). Animals shape their microbiomes in various ways, primarily through host genetics, immunity, and diet (40). Therefore, it is reasonable to hypothesize that nematodes genetically select for a distinct set of microbes within their microbiomes.

### Conclusion

The potential roles of the *B. xylophilus*-associated microbiome have been recognized for some time, but mainly because of the variation in nematode microbiomes across different locations, microbiomes of nematodes from different sites may have different effects. The impact of PWD on trees’ endophytic microbial community remains unclear largely because the strongly observed variations are possibly due to the effect of soil properties or other physiochemical parameters related to different sampling locations. Our study showed distinct biogeographic patterns for bacterial and fungal communities associated with nematodes and infested wood samples collected across three vegetation zones in China. While the similarity of the nematode-associated bacterial community was homogenous along geographic distances, the distance decay of PWD pine trees’ bacterial community similarities implies the spatial correlation with environmental variables. Nonetheless, our approaches include network analysis, which provides geographic patterns for co-occurrence and interaction relationships by identifying core and key taxa associated with PWN and PWD wood, which are putative pathogenic microorganisms involved in the disease. Yet further investigations identifying these core microbial species and relevant functional potentials are essential in PWD management.

## MATERIALS AND METHODS

### Pinewood nematode and PWD wood sample collection

Samples were collected from 11 sites, six nematode-only sites and five nematode and wood sites, yielding 34 nematodes and 21 wood samples from Sichuan (SC), Chongqing (CQ), Guangxi (GX), Hunan (HN), Hubei (HB), Shaanxi (SN), Anhui (AH), Jiangsu (JS), Zhejiang (ZJ), Jilin (JL) province, and the United States (Fig. 5; Table S1). The majority of nematode samples were sourced from the Center for Biological Disaster Prevention and Control, National Forestry and Grassland Administration of China, though there was a shortage of infested wood samples. Three samples were collected from each site as biological replicates. According to China’s Resource and Environment Science and Data Center, we divided the regions into three vegetation types based on the geographical location with temperate coniferous–broadleaf mixed forest (TCBF), northern subtropical evergreen–deciduous broadleaf mixed forest (NEDBF), and central subtropical evergreen broad-leaved forests (CEBLF) (41, 42). Dry, rosin-without, and blue-stain fungus-infested host pine trees were selected from PWN-infected areas, sawed into small pieces, and returned in a sample bag stored at −80°C. Several wood blocks were subjected to the modified Baerman funnel technique at room temperature for 24 hours, and the PBS solution (137 mM NaCl, 4.3 mM Na_2_HPO_4_, 2.7 mM KCl, 1.47 mM KH_2_PO_4_; pH 7.4) (43) was added until the liquid level was above the wood sample. The nematode filtrate was picked up, and then five nematodes with three replicates of samples from each region were picked and placed in 1.5-mL centrifuge tubes frozen in liquid nitrogen and then stored at −80°C. The infested sapwoods of *P. massoniana* with blue-stain fungus were sliced into 1–2 cm pieces, and then about 3 g was put into 50-mL centrifuge tubes for each sample, with three duplicates stored at −80°C until DNA extraction.

### DNA isolation, 16S rRNA gene, and ITS2 region amplification

The extraction method of microbial total DNA of the host pine tree is as follows: 3 g of wood blocks and 1 × PBS were put into a sterilized 50-mL centrifuge tube, ultrasonicated for 30 minutes, and 3 × g shock shaker for 1 hour. Then, wood blocks and other impurities were filtered out with 100-µm cell strainers. The liquid was absorbed into the sterile Petri dish for microscopic examination to remove the nematodes, and then the liquid was filtered with a 0.22-µm filter membrane. Then, DNA was extracted according to the basic process of the FastDNA SPIN Kit for soil (MP Biomedicals, California, USA). The wild PWN DNA was also extracted according to the basic process of the FastDNA SPIN Kit. The quality of DNA was evaluated according to the ratio of 260 nm/ 280 nm and 260 nm/ 230 nm. The microbiome we collected comprises gut and surface microbes.

During sequencing library preparation, we used specific primers with 8 bp barcode appended to both paired ends of the sequence to amplify the V4 region of the 16S rRNA gene for bacteria by the primers 515F (GTGCCAGCMGCCGCGG) and 806R (GGACTACHVGGGTWTCTAAT) and the ITS2 gene for fungi by the primer gITS7 (GTGARTCATCGARTCTTTG) and ITS4(TCCTCCGCTTATTGATATGC) (44–46). All PCRs were conducted in 30-µL reactions that consisted of 15 µL of Phusion High-Fidelity PCR Master Mix (New England Biolabs, Ipswich, MA, USA); 0.2 µM of forward and reverse primers, and about 10 ng template DNA. Thermal cycling consisted of initial denaturation at 98°C for 1 minute, followed by 30 cycles of denaturation at 98°C for 10 seconds, annealing at 50°C for 30 seconds, and elongation at 72°C for 30 seconds, and finally 72°C for 5 minutes. Mix the same volume of 1 × loading buffer (containing SYB Green) with PCR products and operate electrophoresis on 2% agarose gel for detection. Samples with bright main strips between 400 and 450 bp were chosen for further experiments.

### High-throughput Illumina sequencing

The mixture of PCR products was purified with the Gene JET Gel Extraction Kit (Thermo Scientific, Waltham, USA). Those amplicon samples were then submitted to Novogene Co., Ltd. (Beijing, China) for 16S rRNA gene high-throughput sequencing by using NovaSeq PE250 (Illumina, San Diego, USA). Microbiota bioinformatics was performed with USEARCH (47). Raw fastq files are demultiplexed with the corresponding barcode sequences by our developed atlas-utils tool (https://github.com/jameslz/biostack-suits version: 0.0.1). After discarding barcode sequences in each read, low-quality sequences were filtered by Trimmomatic (version 0.38) (48). Paired-end reads were merged using the Usearch fastq mergepairs command with the default parameters (usearch11.0.667, http://drive5.com) (49); Reads were discarded if they could not be merged. The primer sequences would affect further operational taxonomic unit (OTU) table construction. Therefore, merged reads without full primer sequences or with more than two nucleotide mismatches in the primer region were discarded, and the clean merged reads (clean tags) without primer sequences were obtained. The Usearch fastq filter was used to filter all the low-quality sequences in the clean tags with default parameters (fastq maxee 1), and the high-quality sequences of the clean tags were obtained. The obtained high-quality reads were sorted in the non-redundant abundance order using USEARCH fastx uniques. The USEARCH unoise3 algorithm (50) filters the non-redundant sequences and error-correction (denoised) reads with minsize = 8 and generate the zero-radius operational taxonomic unit (ZOTU) table with 100% identity. The phylogenetic assignment of the representative sequences of each ZOTU was determined by the Usearch sintax algorithm (51) with the RDP training set (version v16) 16S rRNA database (other functional gene database can be used, such as nifH sequence database) as the reference database (the confidence threshold is 0.8).

### Microbial diversity analysis

We normalized the ZOTUs to the lowest sequences (74,750 for bacteria and 87,359 for fungi) before microbial diversity calculations and statistical analysis. Moreover, in R (version 4.3.1), the “vegan” package was used to calculate the diversity of different samples based on the ZOTU level (52). We used the Shannon indexes (community diversity) and ACE indexes (community richness) to assess the alpha diversity of both wood and nematodes (53). The analysis of the Kruskal–Wallis (KW) rank-sum test was used to calculate microbial variations between different sites of wood and nematodes. Beta diversities of microorganisms were estimated based on Bray–Curtis distance matrixes between samples. To determine the significance of the difference in community composition between sites, permutational multivariate analysis of variance (PERMANOVA or Adonis) was performed using the “adonis2” function in the “vegan” package in R. All plots were created by the “ggplot2” package in R (54).

### Biomarker screening

Based on the comparsion of microbial community abundances across multiple geographical locations at the genus level, Linear discriminant analysis Effect Size (LEfSe) was applied to help find good biomarkers between different locations (http://galaxy.biobakery.org/). The KW rank-sum test (α  =  0.05) was used in the LEfSe analysis to detect features with significantly different abundances between the specified categories, and this was followed by an linear discriminant analysis (LDA) to estimate the effect size of each differentially abundant feature (logarithmic LDA score > 3.0) (55). The larger the LDA score, the more important the microorganism. Good biomarkers are microbes that have LDA scores greater than 3 and show significant differences in abundance between locations.

### Co-occurrence networks and core species analysis

The co-occurrence networks of nematodes and wood microbial communities were constructed across different regions based on ZOTUs that had a relative abundance greater than 0.5% and were present in more than ten samples. Correlations with Spearman’s correlation coefficients (r) greater than 0.7 and a significance level less than 0.05 were selected to construct a correlation network. Each node represented one ZOTU, and each edge represented a strong and significant correlation between the two nodes. The networks were colored based on modules. Red edges represented the positive interactions, and green edges represented the negative correlations (56).

By classifying the connectivity of each node to identify putative core taxa, node topologies were divided into four categories: module hubs [highly connected nodes within modules, within-module connectivity (*Zi*) >2.5], network hubs [highly connected nodes within entire network, *Zi* >2.5 and among-module connectivity (*Pi*) >0.62)], connectors (nodes that connect modules, *Pi* >0.62), and peripherals (nodes connected in modules with few outside connections, *Zi* <2.5 and *Pi* <0.62) (57, 58). All these analyses were performed using “Hmisc” and “igraph” packages in R and networks were graphed using Gephi-0.10.1 (59, 60).

### Mantel test and latitudinal diversity analysis

The Mantel test was used to assess the correlation between bacterial abundance of PWNs and infected tree with geographical distance. The correlation was calculated using the Mantel command (method is Spearman, 999 random permutations) based on Bray–Curtis distances generated by the “vegan” package (61). As the geographical distance increases, the dissimilarity between communities gradually increases, and the microbial community exhibited a distance decay, an important biogeographic pattern.

To assess differences in alpha diversity across the latitude, we performed linear modules using the function “lm” in R, with the ZOTU counts as the response variable and latitude as the predictor variable. The detail protocol is avaliable from Peng (62).

### Statistical analysis

To estimate the significance of alpha and beta diversity among different sites of nematodes and wood, the analysis of KW rank-sum test and permutational multivariate analysis of variance (PERMANOVA or Adonis) was performed, respectively. The Kruskal–Wallis (KW) rank-sum test was used in the LEfSe analysis to detect features with significantly different abundances between the specified genera. The significance of the Mantel test was calculated by random permutations. The key taxa were composed of widespread taxa, common shared taxa, and enriched taxa. ZOTUs that were present in at least 80% of sampling sites in nematode and wood respectively were considered widespread taxa, wheras ZOTUs were shared with all samples among nematode or wood were called common shared taxa. We considered enriched taxa to consist of ZOTUs that were enriched in temperate coniferous–broadleaf mixed forest and northern subtropical evergreen–deciduous broadleaf central subtropical evergreen broad-leaved forests (using “EdgeR” package in R) (22). Differences with *P* value less than 0.05 were considered significant for all those analyses.

## Data Availability

The data sets that support the findings of this study are available in the supplemental material. The raw sequencing data have been uploaded to the NCBI Sequence Read Archive (SRA), accessible at: PRJNA1117403PRJNA1117403. The script for statistical analyses and the OTU table as a text file are available at https://github.com/1932068325cao/Cao2024_Microbiome.git.

## SUPPLEMENTAL MATERIAL

Supplemental material spectrum.00778-24-s0001.docx Fig. S1 to S6; Tables S1 to S6.

ASM does not own the copyrights to Supplemental Material that may be linked to, or accessed through, an article. The authors have granted ASM a non-exclusive, world-wide license to publish the Supplemental Material files. Please contact the corresponding author directly for reuse.

## References

[1] Futai K. 2013. Pine wood nematode, Bursaphelenchus xylophilus. Annu Rev Phytopathol 51:61–83. doi:10.1146/annurev-phyto-081211-172910 23663004

[2] Meng WJ, Li YL, Qu ZL, Zhang YM, Liu B, Liu K, Gao ZW, Dong LN, Sun H. 2024. Fungal community structure shifts in litter degradation along forest succession induced by pine wilt disease. Microbiol Res 280:127588. doi:10.1016/j.micres.2023.127588 38163390

[3] Han X, Li Y, Huang W, Wang R, Hu X, Liang G, Huang S, Lian C, Zhang F, Wu S. 2023. Landscapes drive the dispersal of Monochamus alternatus, vector of the pinewood nematode, revealed by whole-genome resequencing. For Ecol Manage 529:120682. doi:10.1016/j.foreco.2022.120682

[4] Nascimento FX, Espada M, Barbosa P, Rossi MJ, Vicente CSL, Mota M. 2016. Non-specific transient mutualism between the plant parasitic nematode, Bursaphelenchus xylophilus, and the opportunistic bacterium Serratia quinivorans BXF1, a plant-growth promoting pine endophyte with antagonistic effects. Environ Microbiol 18:5265–5276. doi:10.1111/1462-2920.13568 27768814

[5] Vicente CSL, Nascimento F, Espada M, Barbosa P, Mota M, Glick BR, Oliveira S. 2012. Characterization of bacteria associated with pinewood nematode Bursaphelenchus xylophilus. PLoS One 7:e46661. doi:10.1371/journal.pone.0046661 23091599 PMC3473040

[6] Alves M, Pereira A, Vicente C, Matos P, Henriques J, Lopes H, Nascimento F, Mota M, Correia A, Henriques I. 2018. The role of bacteria in pine wilt disease: insights from microbiome analysis. FEMS Microbiol Ecol 94:fiy077. doi:10.1093/femsec/fiy077 29718181

[7] Alves MS, Pereira A, Vicente C, Mota M, Henriques I. 2019. Pseudomonas associated with Bursaphelenchus xylophilus, its insect vector and the host tree: a role in pine wilt disease? For Pathol 49:e12564. doi:10.1111/efp.12564

[8] Zwyssig M, Spescha A, Patt T, Belosevic A, Machado RAR, Regaiolo A, Keel C, Maurhofer M. 2024. Entomopathogenic pseudomonads can share an insect host with entomopathogenic nematodes and their mutualistic bacteria. ISME J 18:wrae028. doi:10.1093/ismejo/wrae028 38381653 PMC10945363

[9] Fu YM, Liu HB, Wu XQ. 2020. Diversity and function of endo-bacteria in Bursaphelenchus xylophilus from Pinus massoniana Lamb. in different regions. Fores 11:487. doi:10.3390/f11050487

[10] An Y, Li Y, Ma L, Li D, Zhang W, Feng Y, Liu Z, Wang X, Wen X, Zhang X. 2022. The changes of microbial communities and key metableolites after early Bursaphelenchus xylophilus invasion of Pinus massoniana. Plants (Basel) 11:2849. doi:10.3390/plants11212849 36365304 PMC9653782

[11] Guo Q, Guo D, Zhao B, Xu J, Li R. 2007. Two cyclic dipeptides from Pseudomonas fluorescens GcM5-1A carried by the pine wood nematode and their toxicities to Japanese black pine suspension cells and seedlings in vitro. J Nematol 39:243–247. 19259494 PMC2586505

[12] Zhang W, Wang X, Li Y, Wei P, Sun N, Wen X, Liu Z, Li D, Feng Y, Zhang X. 2022. Differences between microbial communities of Pinus species having differing level of resistance to the pine wood nematode. Microb Ecol 84:1245–1255. doi:10.1007/s00248-021-01907-4 34757460

[13] Cai S, Jia J, He C, Zeng L, Fang Y, Qiu G, Lan X, Su J, He X. 2021. Multi-omics of pine wood nematode pathogenicity associated with culturable associated microbiota through an artificial assembly approach. Front Plant Sci 12:798539. doi:10.3389/fpls.2021.798539 35046983 PMC8762061

[14] Kawazu K, Zhang H, Yamashita H, Kanzaki H. 1996. Relationship between the pathogenicity of the pine wood nematode, Bursaphelenchus xylophilus, and phenylacetic acid production. Biosci Biotechnol Biochem 60:1413–1415. doi:10.1271/bbb.60.1413 8987588

[15] Kawazu K, Zhang H, Kanzaki H. 1996. Accumulation of benzoic acid in suspension cultured cells of Pinus thunbergii Parl. in response to phenylacetic acid administration. Biosci Biotechnol Biochem 60:1410–1412. doi:10.1271/bbb.60.1410 8987587

[16] Proença DN, Fonseca L, Powers TO, Abrantes IMO, Morais PV. 2014. Diversity of bacteria carried by pinewood nematode in USA and phylogenetic comparison with isolates from other countries. PLoS One 9:e105190. doi:10.1371/journal.pone.0105190 25127255 PMC4134288

[17] Reuter JA, Spacek DV, Snyder MP. 2015. High-throughput sequencing technologies. Mol Cell 58:586–597. doi:10.1016/j.molcel.2015.05.004 26000844 PMC4494749

[18] Du X, Gu S, Zhang Z, Li S, Zhou Y, Zhang Z, Zhang Q, Wang L, Ju Z, Yan C, Li T, Wang D, Yang X, Peng X, Deng Y. 2023. Spatial distribution patterns across multiple microbial taxonomic groups. Environ Res 223:115470. doi:10.1016/j.envres.2023.115470 36775088

[19] Zhao Z, Li H, Sun Y, Shao K, Wang X, Ma X, Hu A, Zhang H, Fan J. 2022. How habitat heterogeneity shapes bacterial and protistan communities in temperate coastal areas near estuaries. Environ Microbiol 24:1775–1789. doi:10.1111/1462-2920.15892 34996132

[20] Bahram M, Hildebrand F, Forslund SK, Anderson JL, Soudzilovskaia NA, Bodegom PM, Bengtsson-Palme J, Anslan S, Coelho LP, Harend H, Huerta-Cepas J, Medema MH, Maltz MR, Mundra S, Olsson PA, Pent M, Põlme S, Sunagawa S, Ryberg M, Tedersoo L, Bork P. 2018. Structure and function of the global topsoil microbiome. Nature New Biol 560:233–237. doi:10.1038/s41586-018-0386-6 30069051

[21] Duan YL, Wang XY, Wang LL, Lian J, Wang WF, Wu FS, Li YL, Li YQ. 2022. Biogeographic patterns of soil microbe communities in the deserts of the Hexi Corridor, Northern China. Catena 211:106026. doi:10.1016/j.catena.2022.106026

[22] Tian H, Zhao L, Koski TM, Sun J. 2022. Microhabitat governs the microbiota of the pinewood nematode and its vector beetle: implication for the prevalence of pine wilt disease. Microbiol Spectr 10:e0078322. doi:10.1128/spectrum.00783-22 35758726 PMC9430308

[23] Proença DN, Grass G, Morais PV. 2017. Understanding pine wilt disease: roles of the pine endophytic bacteria and of the bacteria carried by the disease-causing pinewood nematode. Microbiologyopen 6:e00415. doi:10.1002/mbo3.415 27785885 PMC5387314

[24] Pirttilä AM, Laukkanen H, Pospiech H, Myllylä R, Hohtola A. 2000. Detection of intracellular bacteria in the buds of scotch pine (Pinus sylvestris L.) by in situ hybridization. Appl Environ Microbiol 66:3073–3077. doi:10.1128/AEM.66.7.3073-3077.2000 10877808 PMC92113

[25] Izumi H, Anderson IC, Killham K, Moore ERB. 2008. Diversity of predominant endophytic bacteria in European deciduous and coniferous trees. Can J Microbiol 54:173–179. doi:10.1139/w07-134 18388988

[26] Xue Q, Xiang Y, Wu XQ, Li MJ. 2019. Bacterial communities and virulence associated with pine wood nematode Bursaphelenchus xylophilus from different Pinus spp. Int J Mol Sci 20:3342. doi:10.3390/ijms20133342 31284685 PMC6650965

[27] Guo Y, Lin Q, Chen L, Carballar-Lejarazú R, Zhang A, Shao E, Liang G, Hu X, Wang R, Xu L, Zhang F, Wu S. 2020. Characterization of bacterial communities associated with the pinewood nematode insect vector Monochamus alternatus hope and the host tree Pinus massoniana. BMC Genomics 21:337. doi:10.1186/s12864-020-6718-6 32357836 PMC7195709

[28] Nascimento FX, Hasegawa K, Mota M, Vicente CSL. 2015. Bacterial role in pine wilt disease development-review and future perspectives. Environ Microbiol Rep 7:51–63. doi:10.1111/1758-2229.12202 25139220

[29] Wang X, Wu H, Dai C, Wang X, Wang L, Xu J, Lu Z. 2022. Microbial interactions enhanced environmental fitness and expanded ecological niches under dibutyl phthalate and cadmium co-contamination. Environ Pollut 306:119362. doi:10.1016/j.envpol.2022.119362 35489538

[30] de Oliveira TC, Freyria NJ, Sarmiento-Villamil JL, Porth I, Tanguay P, Bernier L. 2024. Unraveling the transcriptional features and gene expression networks of pathogenic and saprotrophic Ophiostoma species during the infection of Ulmus americana. Microbiol Spectr 12:e0369423. doi:10.1128/spectrum.03694-23 38230934 PMC10845970

[31] Blackwell M. 2017. Made for each other: Ascomycete yeasts and insects. Microbiol Spectr 5:spectrum. doi:10.1128/microbiolspec.FUNK-0081-2016 PMC11687504 28597823

[32] Chai VZ, Farajzadeh T, Meng Y, Lo SB, Asaed TA, Taylor CJ, Glater EE. 2024. Chemical basis of microbiome preference in the nematode C. elegans. Sci Rep 14:1350. doi:10.1038/s41598-024-51533-6 38228683 PMC10791660

[33] Dirksen P, Marsh SA, Braker I, Heitland N, Wagner S, Nakad R, Mader S, Petersen C, Kowallik V, Rosenstiel P, Félix MA, Schulenburg H. 2016. The native microbiome of the nematode Caenorhabditis elegans: gateway to a new host-microbiome model. BMC Biol 14:38. doi:10.1186/s12915-016-0258-1 27160191 PMC4860760

[34] Berg M, Stenuit B, Ho J, Wang A, Parke C, Knight M, Alvarez-Cohen L, Shapira M. 2016. Assembly of the Caenorhabditis elegans gut microbiota from diverse soil microbial environments. ISME J 10:1998–2009. doi:10.1038/ismej.2015.253 26800234 PMC5029150

[35] Wu L, Zhang Y, Guo X, Ning D, Zhou X, Feng J, Yuan MM, Liu S, Guo J, Gao Z, Ma J, Kuang J, Jian S, Han S, Yang Z, Ouyang Y, Fu Y, Xiao N, Liu X, Wu L, Zhou A, Yang Y, Tiedje JM, Zhou J. 2022. Reduction of microbial diversity in grassland soil is driven by long-term climate warming. Nat Microbiol 7:1054–1062. doi:10.1038/s41564-022-01147-3 35697795

[36] Guo X, Endler A, Poll C, Marhan S, Ruess L. 2021. Independent effects of warming and altered precipitation pattern on nematode community structure in an arable field. Agric Ecosyst Environ 316:107467. doi:10.1016/j.agee.2021.107467

[37] Hao X, Liu X, Chen J, Wang B, Li Y, Ye Y, Ma W, Ma L. 2022. Effects on community composition and function Pinus massoniana infected by Bursaphelenchus xylophilus. BMC Microbiol 22:1–16. doi:10.1186/s12866-022-02569-z 35690728 PMC9188149

[38] Monroy F, van der Putten WH, Yergeau E, Mortimer SR, Duyts H, Bezemer TM. 2012. Community patterns of soil bacteria and nematodes in relation to geographic distance. Soil Biol Biochem 45:1–7. doi:10.1016/j.soilbio.2011.10.006

[39] Lo WS, Han Z, Witte H, Röseler W, Sommer RJ. 2022. Synergistic interaction of gut microbiota enhances the growth of nematode through neuroendocrine signaling. Curr Biol 32:2037–2050. doi:10.1016/j.cub.2022.03.056 35397201

[40] Lynch JB, Hsiao EY. 2019. Microbiomes as sources of emergent host phenotypes. Science 365:1405–1409. doi:10.1126/science.aay0240 31604267

[41] Zhou B, Sterck F, Kruijt B, Fan ZX, Zuidema PA. 2023. Diel and seasonal stem growth responses to climatic variation are consistent across species in a subtropical tree community. New Phytol 240:2253–2264. doi:10.1111/nph.19275 37737019

[42] Wang H, Ding Y, Zhang Y, Wang J, Freedman ZB, Liu P, Cong W, Wang J, Zang R, Liu S. 2023. Evenness of soil organic carbon chemical components changes with tree species richness, composition and functional diversity across forests in China. Glob Chang Biol 29:2852–2864. doi:10.1111/gcb.16653 36840370

[43] Li H, Dietrich C, Zhu N, Mikaelyan A, Ma B, Pi R, Liu Y, Yang M, Brune A, Mo J. 2016. Age polyethism drives community structure of the bacterial gut microbiota in the fungus-cultivating termite Odontotermes formosanus. Environ Microbiol 18:1440–1451. doi:10.1111/1462-2920.13046 26346907

[44] Zhao BG, Lin F. 2005. Mutualistic symbiosis between Bursaphelenchus xylophilus and bacteria of the genus Pseudomonas. For Pathol 35:339–345. doi:10.1111/j.1439-0329.2005.00417.x

[45] Kostovcik M, Bateman CC, Kolarik M, Stelinski LL, Jordal BH, Hulcr J. 2015. The ambrosia symbiosis is specific in some species and promiscuous in others: evidence from community pyrosequencing. ISME J 9:126–138. doi:10.1038/ismej.2014.115 25083930 PMC4274425

[46] Gao M, Xiong C, Gao C, Tsui CKM, Wang M-M, Zhou X, Zhang AM, Cai L. 2021. Disease-induced changes in plant microbiome assembly and functional adaptation. Microbiome 9:187. doi:10.1186/s40168-021-01138-2 34526096 PMC8444440

[47] Edgar RC. 2010. Search and clustering orders of magnitude faster than BLAST. Bioinformatics 26:2460–2461. doi:10.1093/bioinformatics/btq461 20709691

[48] Bolger AM, Lohse M, Usadel B. 2014. Trimmomatic: a flexible trimmer for Illumina sequence data. Bioinformatics 30:2114–2120. doi:10.1093/bioinformatics/btu170 24695404 PMC4103590

[49] Edgar RC, Flyvbjerg H. 2015. Error filtering, pair assembly and error correction for next-generation sequencing reads. Bioinformatics 31:3476–3482. doi:10.1093/bioinformatics/btv401 26139637

[50] Edgar RC. 2016. UNOISE2: improved error-correction for Illumina 16S and ITS amplicon sequencing. bioRxiv. doi:10.1101/081257

[51] Edgar RC. 2016. SINTAX: a simple non-Bayesian taxonomy classifier for 16S and ITS sequences. Bioinformatics. doi:10.1101/074161

[52] Oksanen J, Simpson G, Blanchet FG, Kindt R, Legendre P, Minchin P, Hara P, Solymos P, Stevens H, Szöcs E, Wagner H, Barbour M, Bedward M, Bolker B, Borcard D, Carvalho G, Chirico M, De Cáceres M, Durand S, Weedon J. 2022. Vegan community ecology, R package version 2.6-4. Available from: https://CRAN.R-project.org/package=vegan

[53] Shannon CE. 1948. A mathematical theory of communication. Bell Syst Tech J 27:379–423. doi:10.1002/j.1538-7305.1948.tb01338.x

[54] Wickham H. 2016. ggplot2: elegant graphics for data analysis. Springer-Verlag New York.

[55] Segata N, Izard J, Waldron L, Gevers D, Miropolsky L, Garrett WS, Huttenhower C. 2011. Metagenomic biomarker discovery and explanation. Genome Biol 12:R60. doi:10.1186/gb-2011-12-6-r60 21702898 PMC3218848

[56] Fan K, Cardona C, Li Y, Shi Y, Xiang X, Shen C, Wang H, Gilbert JA, Chu H. 2017. Rhizosphere-associated bacterial network structure and spatial distribution differ significantly from bulk soil in wheat crop fields. Soil Biol Biochem 113:275–284. doi:10.1016/j.soilbio.2017.06.020

[57] Deng Y, Jiang Y-H, Yang Y, He Z, Luo F, Zhou J. 2012. Molecular ecological network analyses. BMC Bioinformatics 13:113. doi:10.1186/1471-2105-13-113 22646978 PMC3428680

[58] Shi S, Nuccio EE, Shi ZJ, He Z, Zhou J, Firestone MK, Johnson N. 2016. The interconnected rhizosphere: high network complexity dominates rhizosphere assemblages. Ecol Lett 19:926–936. doi:10.1111/ele.12630 27264635

[59] Harrell Jr FE. 2013. Hmisc: harrell miscellaneous, R package version 5.1-1. Available from: https://CRAN.R-project.org/package=Hmisc

[60] Csárdi G, Nepusz T, Traag V, Horvát S, Zanini F, Noom D, Müller K. 2023. igraph: network analysis and visualization in R, R package version 1.5.1. Available from: https://CRAN.R-project.org/package=igraph

[61] Zhou ZB, Zhang YJ, Zhang FG. 2022. Abundant and rare bacteria possess different diversity and function in crop monoculture and rotation systems across regional farmland. Soil Biol Biochem 171:108742. doi:10.1016/j.soilbio.2022.108742

[62] Peng ZH, Jiao S, Wei GH. 2021. Research methods of microbial biogeography. Bio-101:e2003930. doi:10.21769/BioProtoc.2003930

