# Supplementary material for: Distinct biogeographic patterns for bacteria and fungi in association with Bursaphelenchus xylophilus nematodes and infested pinewood
Source: Microbiol Spectr. 2024 Aug 20;12(10):e00778-24. doi: 10.1128/spectrum.00778-24 (PMC11448397; doi:10.1128/spectrum.00778-24)
Supplement: Supplemental material — Fig. S1 to S6; Tables S1 to S6. [file spectrum.00778-24-s0001.docx]

**SUPPLEMENTARY FIGURES AND TABLES**


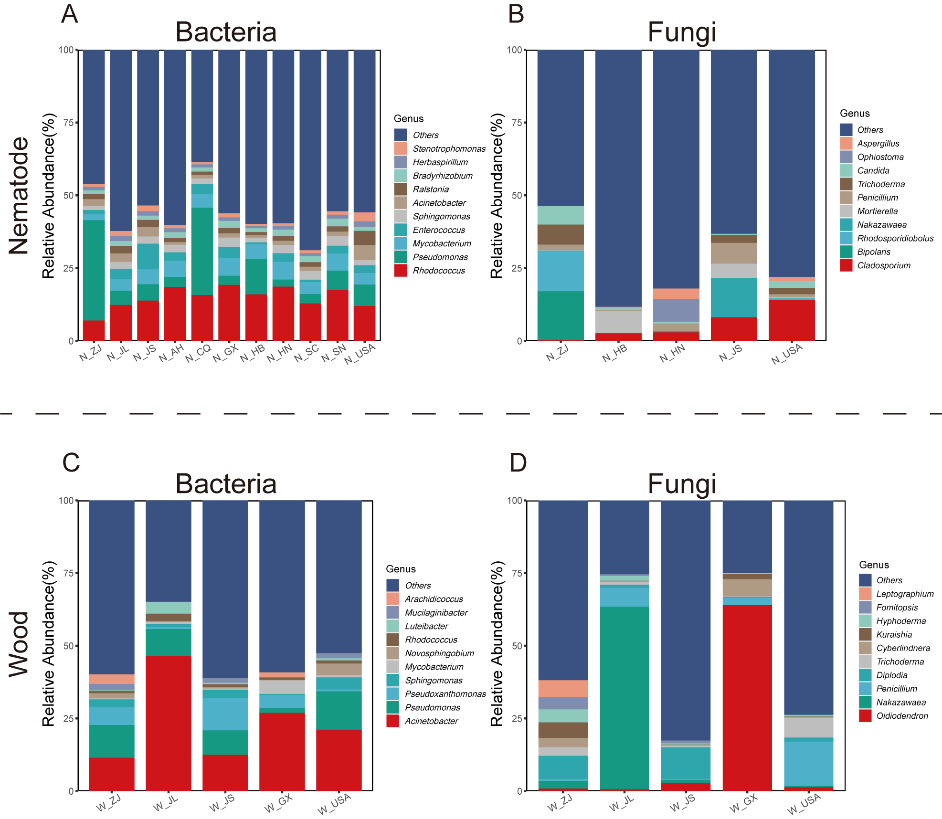


**FIG S1** Bacterial and fungal composition at genus level of PWNs and host pine trees from diverse regions. (A) The top 10 genera of bacteria, and (B) the top 10 genera of fungi of PWNs in relative abundance. (C) The top 10 genera of bacteria, and (D) the top 10 genera of fungi of host pine trees in relative abundance.


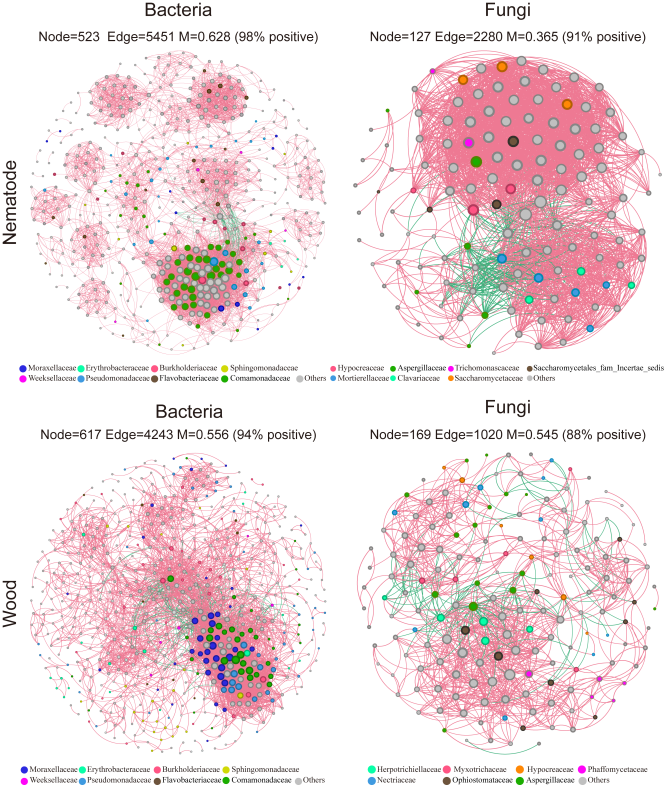


**FIG S2** Interaction networks between ZOTUs in nematodes and wood, using data for ZOTUs with relative abundance > 0.5%. Each node represents one ZOTU, and each edge represents a strong and significant correlation (r > 0.7, *P* < 0.05) between two nodes. The red edge represents the positive interactions, and the green edge represents the negative correlations. The nodes are colored by family. The capital letter M means modularity, an index measuring the extent to which a network is divided into modules.


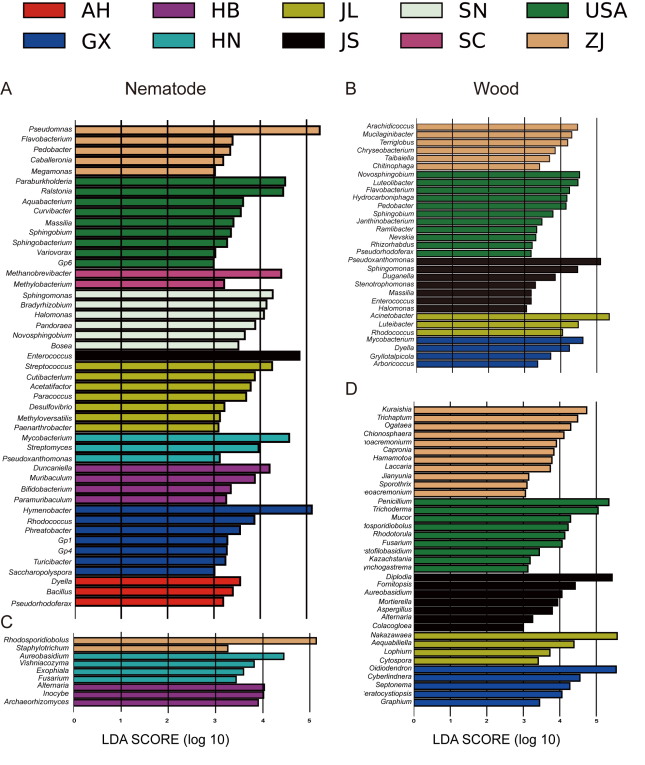


**FIG S3** Taxonomic differences of nematode and wood associated microbiota from diverse sites. Linear discriminant analysis (LDA) effect size (LEfSe) analysis indicated significant fungal differences between different sites. LDA scores (log10) > 3 and *P* < 0.05 are shown. (A) the bacterial differences of nematode, (C) the fungal differences of nematode, (B) the bacterial differences of wood, (D) the fungal differences of wood.


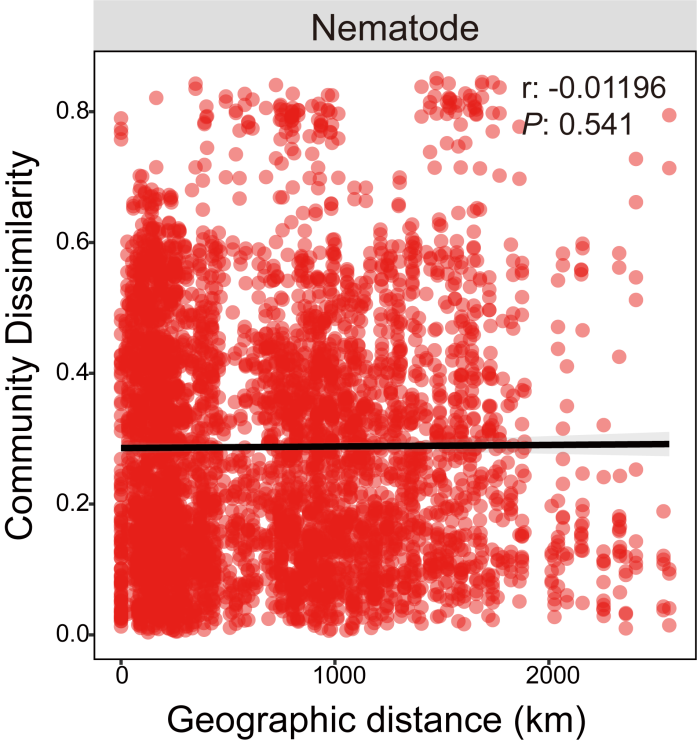


**FIG S4** Relationships between the Bray-Curtis dissimilarities of PWNs from 33 sampling sites except USA.


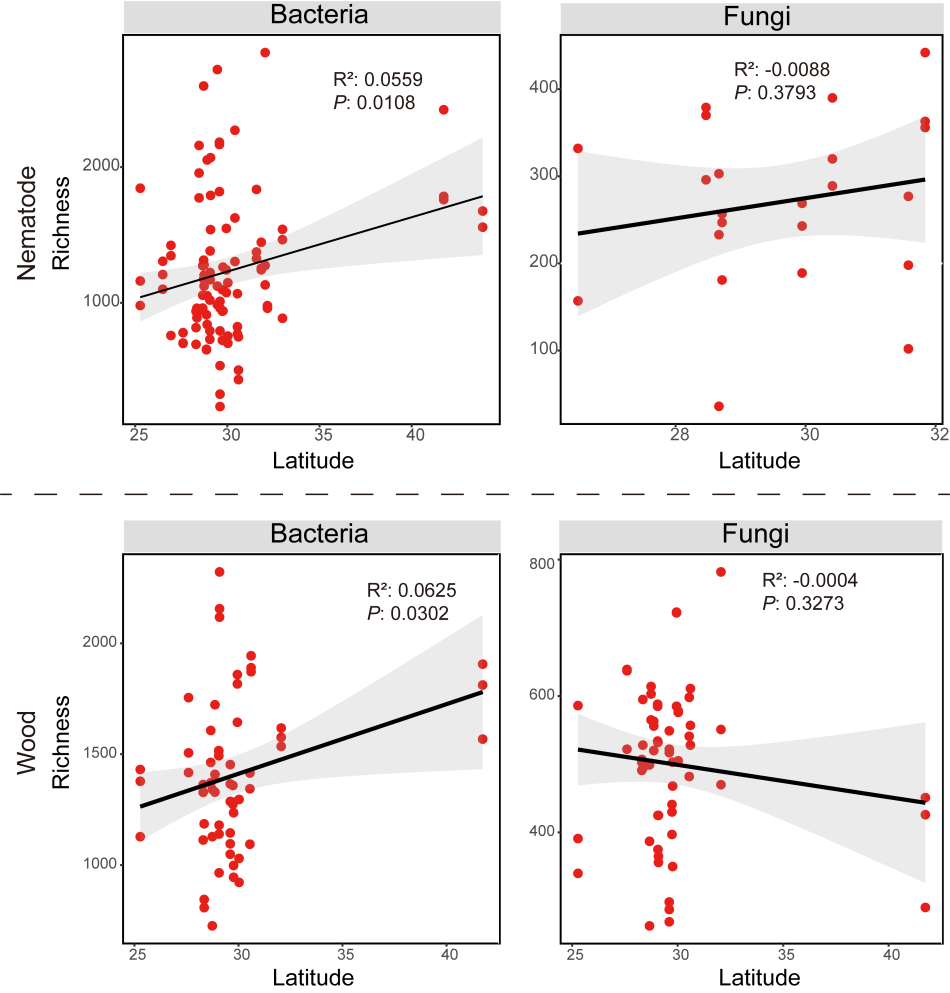


**FIG S5** The differences in richness index across the latitude.The lines represent the linear regression results. The shaded areas show the 95% confidence interval.


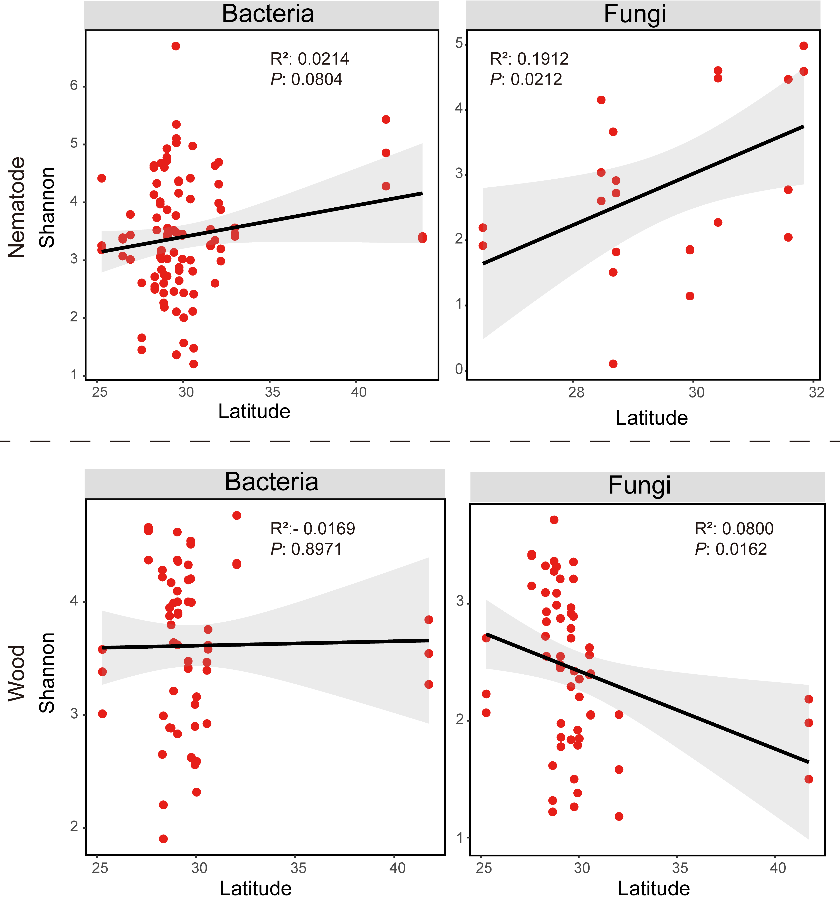


**Fig. S6** Shannon index of microbes vary with latitude gradient. The y axis represents the observed ZOTUs for bacterial and fungal community. The x axis of shows the latitude gradient.

**TABLE S1** Details information of sampling latitude, longitude, location, group and vegetation type.

| **Sample** | **Sample numbers (N)** | **Latitude** | **Longitude** | **Location** | **Group** | **Vegetation type** |
| --- | --- | --- | --- | --- | --- | --- |
| N_GX_G | 3 | 25.277770N | 110.319858E | GX (Guilin) | Nematode | Central subtropical evergreen broad-leaved forests |
| N_HN_Y | 3 | 26.497317N | 111.473538E | HN (Yongzhou) | Nematode | Central subtropical evergreen broad-leaved forests |
| N_HN_H | 3 | 26.940537N | 112.702888E | HN (Hengyang) | Nematode | Central subtropical evergreen broad-leaved forests |
| N_HN_C | 3 | 28.467363N | 113.191082E | HN (Changsha) | Nematode | Central subtropical evergreen broad-leaved forests |
| N_HN_M | 3 | 28.714631N | 109.656878E | HN (Xiangxi) | Nematode | Central subtropical evergreen broad-leaved forests |
| N_ZJ_WP | 3 | 27.599036N | 120.565180E | ZJ (Wenzhou) | Nematode | Central subtropical evergreen broad-leaved forests |
| N_ZJ_LL | 3 | 28.301513N | 119.759631E | ZJ (Lishui) | Nematode | Central subtropical evergreen broad-leaved forests |
| N_ZJ_WY | 3 | 28.350045N | 120.495603E | ZJ (Wenzhou) | Nematode | Central subtropical evergreen broad-leaved forests |
| N_ZJ_LJ | 3 | 28.666482N | 120.098057E | ZJ (Lishui) | Nematode | Central subtropical evergreen broad-leaved forests |
| N_ZJ_QJ | 3 | 28.739902N | 118.628868E | ZJ (Quzhou) | Nematode | Central subtropical evergreen broad-leaved forests |
| N_ZJ_TL | 3 | 28.868580N | 121.159365E | ZJ (Taizhou) | Nematode | Central subtropical evergreen broad-leaved forests |
| N_ZJ_J | 3 | 28.906128N | 120.356298E | ZJ (Jinhua) | Nematode | Central subtropical evergreen broad-leaved forests |
| N_ZJ_JP | 3 | 29.051573N | 120.692892E | ZJ (Jinhua) | Nematode | Central subtropical evergreen broad-leaved forests |
| N_ZJ_JW | 3 | 29.067173N | 119.490296E | ZJ (Jinhua) | Nematode | Central subtropical evergreen broad-leaved forests |
| N_ZJ_TT | 3 | 29.089241N | 120.908386E | ZJ (Taizhou) | Nematode | Central subtropical evergreen broad-leaved forests |
| N_ZJ_SS | 3 | 29.601953N | 120.839856E | ZJ (Shaoxing) | Nematode | Central subtropical evergreen broad-leaved forests |
| N_ZJ_HC | 3 | 29.604013N | 119.039692E | ZJ (Hangzhou) | Nematode | Central subtropical evergreen broad-leaved forests |
| N_ZJ_NY | 3 | 29.731007N | 121.601538E | ZJ (Ningbo) | Nematode | Central subtropical evergreen broad-leaved forests |
| N_ZJ_HT | 3 | 29.763235N | 119.697551E | ZJ (Hangzhou) | Nematode | Central subtropical evergreen broad-leaved forests |
| N_ZJ_NB | 3 | 29.945760N | 121.783000E | ZJ (Ningbo) | Nematode | Central subtropical evergreen broad-leaved forests |
| N_ZJ_ZD | 3 | 30.027061N | 122.120957E | ZJ (Zhoushan) | Nematode | Central subtropical evergreen broad-leaved forests |
| N_ZJ_HD | 3 | 30.547633N | 119.983469E | ZJ (Huzhou) | Nematode | Central subtropical evergreen broad-leaved forests |
| N_CQ_W | 3 | 29.458595N | 107.384571E | CQ (Wulong) | Nematode | Central subtropical evergreen broad-leaved forests |
| N_SC_L | 3 | 29.571660N | 103.790178E | SC (Leshan) | Nematode | Central subtropical evergreen broad-leaved forests |
| N_ZJ_HA | 3 | 30.604284N | 119.660742E | ZJ (Huzhou) | Nematode | Central subtropical evergreen broad-leaved forests |
| N_HB_Y | 3 | 30.412235N | 111.241355E | HB (Yichang) | Nematode | Central subtropical evergreen broad-leaved forests |
| N_HB_X | 3 | 31.839266N | 111.690787E | HB (Xiangyang) | Nematode | Northern subtropical evergreen-deciduous broadleaf |
| N_JS_W | 3 | 31.581253N | 120.093817E | JS (Wuxi) | Nematode | Northern subtropical evergreen-deciduous broadleaf |
| N_JS_NX | 3 | 32.051488N | 118.863950E | JS (Nanjing) | Nematode | Northern subtropical evergreen-deciduous broadleaf |
| N_AH_C | 3 | 32.175592N | 118.189209E | AH (Chuzhou) | Nematode | Northern subtropical evergreen-deciduous broadleaf |
| N_SN_X | 3 | 32.990609N | 107.772820E | SN (Xixiang) | Nematode | Northern subtropical evergreen-deciduous broadleaf |
| N_JL_T | 3 | 41.737000N | 125.947211E | JL (Tonghua) | Nematode | Temperate coniferous-broadleaf mixed forest |
| N_JL_J | 2 | 43.848998N | 126.533915E | JL (Jilin) | Nematode | Temperate coniferous-broadleaf mixed forest |
| N_USA | 3 | unknown | unknown | USA | Nematode | - |
| W_GX_G | 3 | 25.277770N | 110.319858E | GX (Guilin) | Wood | Central subtropical evergreen broad-leaved forests |
| W_ZJ_WP | 3 | 27.599036N | 120.565180E | ZJ (Wenzhou) | Wood | Central subtropical evergreen broad-leaved forests |
| W_ZJ_LL | 3 | 28.301513N | 119.759631E | ZJ (Lishui) | Wood | Central subtropical evergreen broad-leaved forests |
| W_ZJ_WY | 3 | 28.350045N | 120.495603E | ZJ (Wenzhou) | Wood | Central subtropical evergreen broad-leaved forests |
| W_ZJ_LJ | 3 | 28.666482N | 120.098057E | ZJ (Lishui) | Wood | Central subtropical evergreen broad-leaved forests |
| W_ZJ_QJ | 3 | 28.739902N | 118.628868E | ZJ (Quzhou) | Wood | Central subtropical evergreen broad-leaved forests |
| W_ZJ_TL | 3 | 28.868580N | 121.159365E | ZJ (Taizhou) | Wood | Central subtropical evergreen broad-leaved forests |
| W_ZJ_JP | 3 | 29.051573N | 120.692892E | ZJ (Jinhua) | Wood | Central subtropical evergreen broad-leaved forests |
| W_ZJ_JW | 3 | 29.067173N | 119.490296E | ZJ (Jinhua) | Wood | Central subtropical evergreen broad-leaved forests |
| W_ZJ_TT | 3 | 29.089241N | 120.908386E | ZJ (Taizhou) | Wood | Central subtropical evergreen broad-leaved forests |
| W_ZJ_SS | 3 | 29.601953N | 120.839856E | ZJ (Shaoxing) | Wood | Central subtropical evergreen broad-leaved forests |
| W_ZJ_HC | 3 | 29.604013N | 119.039692E | ZJ (Hangzhou) | Wood | Central subtropical evergreen broad-leaved forests |
| W_ZJ_NY | 3 | 29.731007N | 121.601538E | ZJ (Ningbo) | Wood | Central subtropical evergreen broad-leaved forests |
| W_ZJ_HT | 3 | 29.763235N | 119.697551E | ZJ (Hangzhou) | Wood | Central subtropical evergreen broad-leaved forests |
| W_ZJ_NB | 3 | 29.945760N | 121.783000E | ZJ (Ningbo) | Wood | Central subtropical evergreen broad-leaved forests |
| W_ZJ_ZD | 3 | 30.027061N | 122.120957E | ZJ (Zhoushan) | Wood | Central subtropical evergreen broad-leaved forests |
| W_ZJ_HD | 3 | 30.547633N | 119.983469E | ZJ (Huzhou) | Wood | Central subtropical evergreen broad-leaved forests |
| W_ZJ_HA | 3 | 30.604284N | 119.660742E | ZJ (Huzhou) | Wood | Central subtropical evergreen broad-leaved forests |
| W_JS_NX | 3 | 32.051488N | 118.863950E | JS (Nanjing) | Wood | Northern subtropical evergreen-deciduous broadleaf |
| W_JL_T | 3 | 41.737000N | 125.947211E | JL (Tonghua) | Wood | Temperate coniferous-broadleaf mixed forest |
| W_USA | 3 | unknown | unknown | USA | Wood | - |

**TABLE S2** Detailed taxonomic information for module hubs and connectors within nematode bacterial and fungal network.

| **Nodes-id** | **Phylum** | **Class** | **Order** | **Family** | **Genus** | **Zi** | **Pi** | **Type** |
| --- | --- | --- | --- | --- | --- | --- | --- | --- |
| ZOTU_4740 | Pseudomonadota | Betaproteobacteria | Burkholderiales | unclassified | *unclassified* | 2.48 | 0.65 | Connectors |
| ZOTU_126 | Bacteroidota | Flavobacteriia | Flavobacteriales | Flavobacteriaceae | *Flavobacterium* | 0.32 | 0.68 | Connectors |
| ZOTU_19 | Pseudomonadota | Betaproteobacteria | Burkholderiales | Comamonadaceae | *unclassified* | 0.73 | 0.68 | Connectors |
| ZOTU_2 | Pseudomonadota | Betaproteobacteria | Burkholderiales | Comamonadaceae | *unclassified* | 0.77 | 0.69 | Connectors |
| ZOTU_25 | Pseudomonadota | Betaproteobacteria | Burkholderiales | Burkholderiaceae | *Pandoraea* | 1.24 | 0.69 | Connectors |
| ZOTU_8608 | Pseudomonadota | Gammaproteobacteria | Pseudomonadales | unclassified | *unclassified* | 0.18 | 0.69 | Connectors |
| ZOTU_9908 | Pseudomonadota | Betaproteobacteria | Burkholderiales | Comamonadaceae | *unclassified* | 0.73 | 0.69 | Connectors |
| ZOTU_7 | Actinomycetota | Actinomycetota | Mycobacteriales | Mycobacteriaceae | *Mycobacterium* | 1.73 | 0.69 | Connectors |
| ZOTU_10072 | Pseudomonadota | Betaproteobacteria | Burkholderiales | Comamonadaceae | *unclassified* | 0.00 | 0.75 | Connectors |
| ZOTU_1018 | unclassified | unclassified | unclassified | unclassified | *unclassified* | 0.65 | 0.75 | Connectors |
| ZOTU_11060 | Actinomycetota | Actinomycetota | Mycobacteriales | Mycobacteriaceae | *Mycobacterium* | 0.00 | 0.75 | Connectors |
| ZOTU_12751 | Actinomycetota | Actinomycetota | Mycobacteriales | Nocardiaceae | *Rhodococcus* | -0.40 | 0.75 | Connectors |
| ZOTU_133 | Pseudomonadota | Alphaproteobacteria | Sphingomonadales | Erythrobacteraceae | *Novosphingobium* | -0.28 | 0.75 | Connectors |
| ZOTU_13515 | Pseudomonadota | Betaproteobacteria | Burkholderiales | Comamonadaceae | *unclassified* | -0.28 | 0.75 | Connectors |
| ZOTU_146 | Pseudomonadota | Alphaproteobacteria | Rhizobiales | Phreatobacteraceae | *Phreatobacter* | -0.28 | 0.75 | Connectors |
| ZOTU_14669 | Pseudomonadota | Gammaproteobacteria | Pseudomonadales | unclassified | *unclassified* | 0.87 | 0.75 | Connectors |
| ZOTU_1647 | Pseudomonadota | Gammaproteobacteria | Pseudomonadales | Pseudomonadaceae | *unclassified* | -0.08 | 0.75 | Connectors |
| ZOTU_197 | Pseudomonadota | unclassified | unclassified | unclassified | *unclassified* | -0.40 | 0.75 | Connectors |
| ZOTU_198 | Pseudomonadota | Betaproteobacteria | Burkholderiales | Comamonadaceae | *Aquabacterium* | 0.00 | 0.75 | Connectors |
| ZOTU_199 | Bacteroidota | Flavobacteriia | Flavobacteriales | Flavobacteriaceae | *unclassified* | 0.00 | 0.75 | Connectors |
| ZOTU_20 | Pseudomonadota | Betaproteobacteria | Burkholderiales | Burkholderiaceae | *Ralstonia* | -0.93 | 0.75 | Connectors |
| ZOTU_215 | Bacteroidota | Bacteroidia | Bacteroidales | Muribaculaceae | *unclassified* | 0.87 | 0.75 | Connectors |
| ZOTU_225 | Pseudomonadota | Alphaproteobacteria | Sphingomonadales | Sphingomonadaceae | *Sphingobium* | -0.28 | 0.75 | Connectors |
| ZOTU_247 | Pseudomonadota | Gammaproteobacteria | Pseudomonadales | Pseudomonadaceae | *Pseudomonas* | -0.28 | 0.75 | Connectors |
| ZOTU_282 | Pseudomonadota | Alphaproteobacteria | Rhizobiales | unclassified | *unclassified* | 0.18 | 0.75 | Connectors |
| ZOTU_3 | Actinomycetota | Actinomycetota | Mycobacteriales | Nocardiaceae | *Rhodococcus* | -0.28 | 0.75 | Connectors |
| ZOTU_3030 | Pseudomonadota | Betaproteobacteria | unclassified | unclassified | *unclassified* | -0.28 | 0.75 | Connectors |
| ZOTU_3398 | Bacillota | Erysipelotrichia | Erysipelotrichales | unclassified | *unclassified* | 0.00 | 0.75 | Connectors |
| ZOTU_3729 | Pseudomonadota | Betaproteobacteria | Burkholderiales | unclassified | *unclassified* | 0.18 | 0.75 | Connectors |
| ZOTU_379 | Pseudomonadota | Betaproteobacteria | unclassified | unclassified | *unclassified* | 0.23 | 0.75 | Connectors |
| ZOTU_3850 | Pseudomonadota | Gammaproteobacteria | unclassified | unclassified | *unclassified* | 1.15 | 0.75 | Connectors |
| ZOTU_398 | Pseudomonadota | Gammaproteobacteria | Pseudomonadales | Moraxellaceae | *Acinetobacter* | 0.23 | 0.75 | Connectors |
| ZOTU_437 | Ignavibacteriae | Ignavibacteria | Ignavibacteriales | Ignavibacteriaceae | *Ignavibacterium* | 0.21 | 0.75 | Connectors |
| ZOTU_445 | Bacteroidota | Bacteroidia | Bacteroidales | Muribaculaceae | *Duncaniella* | 0.87 | 0.75 | Connectors |
| ZOTU_4641 | Pseudomonadota | Betaproteobacteria | Burkholderiales | Comamonadaceae | *unclassified* | -0.28 | 0.75 | Connectors |
| ZOTU_467 | Pseudomonadota | Gammaproteobacteria | Alteromonadales | Alteromonadaceae | *unclassified* | 0.23 | 0.75 | Connectors |
| ZOTU_576 | Euryarchaeota | Methanobacteria | Methanobacteriales | Methanobacteriaceae | *Methanobrevibacter* | 0.00 | 0.75 | Connectors |
| ZOTU_5901 | Actinomycetota | Actinomycetota | Mycobacteriales | unclassified | *unclassified* | -1.28 | 0.75 | Connectors |
| ZOTU_601 | Bacteroidota | Bacteroidia | Bacteroidales | Prevotellaceae | *Prevotella* | 0.00 | 0.75 | Connectors |
| ZOTU_627 | Bacteroidota | Bacteroidia | Bacteroidales | Rikenellaceae | *Alistipes* | 1.15 | 0.75 | Connectors |
| ZOTU_6538 | Pseudomonadota | Betaproteobacteria | Burkholderiales | Comamonadaceae | *unclassified* | 0.18 | 0.75 | Connectors |
| ZOTU_674 | Bacillota | Clostridia | Clostridiales | Lachnospiraceae | *unclassified* | 0.00 | 0.75 | Connectors |
| ZOTU_707 | Bacillota | Clostridia | Clostridiales | Lachnospiraceae | *unclassified* | 1.15 | 0.75 | Connectors |
| ZOTU_7096 | Pseudomonadota | Betaproteobacteria | Burkholderiales | Comamonadaceae | *unclassified* | -0.40 | 0.75 | Connectors |
| ZOTU_716 | Bacillota | Clostridia | Clostridiales | Ruminococcaceae | *unclassified* | 1.15 | 0.75 | Connectors |
| ZOTU_7206 | Pseudomonadota | Gammaproteobacteria | unclassified | unclassified | *unclassified* | 0.87 | 0.75 | Connectors |
| ZOTU_7262 | Pseudomonadota | Betaproteobacteria | Burkholderiales | unclassified | *unclassified* | -1.28 | 0.75 | Connectors |
| ZOTU_73 | Pseudomonadota | Betaproteobacteria | Burkholderiales | Comamonadaceae | *Aquabacterium* | 0.00 | 0.75 | Connectors |
| ZOTU_750 | Bacteroidota | Bacteroidia | Bacteroidales | Bacteroidaceae | *Bacteroides* | 0.65 | 0.75 | Connectors |
| ZOTU_754 | Bacteroidota | Flavobacteriia | Flavobacteriales | Weeksellaceae | *unclassified* | -0.80 | 0.75 | Connectors |
| ZOTU_7932 | Pseudomonadota | Betaproteobacteria | Burkholderiales | Comamonadaceae | *unclassified* | -0.93 | 0.75 | Connectors |
| ZOTU_801 | Bacillota | Bacilli | Bacillales | Staphylococcaceae | *Staphylococcus* | 0.65 | 0.75 | Connectors |
| ZOTU_802 | Bacillota | Bacilli | Lactobacillales | unclassified | *unclassified* | 0.00 | 0.75 | Connectors |
| ZOTU_8153 | Pseudomonadota | unclassified | unclassified | unclassified | *unclassified* | -0.28 | 0.75 | Connectors |
| ZOTU_825 | Bacillota | Clostridia | Clostridiales | Lachnospiraceae | *unclassified* | 0.41 | 0.75 | Connectors |
| ZOTU_8253 | Actinomycetota | Actinomycetota | Mycobacteriales | Nocardiaceae | *Rhodococcus* | 0.00 | 0.75 | Connectors |
| ZOTU_826 | Bacillota | Clostridia | Clostridiales | unclassified | *unclassified* | 0.00 | 0.75 | Connectors |
| ZOTU_8673 | Pseudomonadota | Betaproteobacteria | Burkholderiales | unclassified | *unclassified* | 0.00 | 0.75 | Connectors |
| ZOTU_889 | Bacteroidota | Bacteroidia | Bacteroidales | Porphyromonadaceae | *Parabacteroides* | 0.41 | 0.75 | Connectors |
| ZOTU_901 | Bacteroidota | Flavobacteriia | Flavobacteriales | Flavobacteriaceae | *unclassified* | 0.00 | 0.75 | Connectors |
| ZOTU_907 | Bacteroidota | unclassified | unclassified | unclassified | *unclassified* | 1.15 | 0.75 | Connectors |
| ZOTU_9398 | unclassified | unclassified | unclassified | unclassified | *unclassified* | -0.40 | 0.75 | Connectors |
| ZOTU_941 | Actinomycetota | Actinomycetota | Mycobacteriales | Nocardiaceae | *Rhodococcus* | -0.40 | 0.75 | Connectors |
| ZOTU_953 | Bacillota | Bacilli | Lactobacillales | Lactobacillaceae | *unclassified* | 1.15 | 0.75 | Connectors |
| ZOTU_96 | unclassified | unclassified | unclassified | unclassified | *unclassified* | 0.23 | 0.75 | Connectors |
| ZOTU_969 | Bacillota | Clostridia | Clostridiales | Ruminococcaceae | *unclassified* | 0.65 | 0.75 | Connectors |
| ZOTU_9706 | Pseudomonadota | Gammaproteobacteria | unclassified | unclassified | *unclassified* | 0.00 | 0.75 | Connectors |
| ZOTU_983 | Bacillota | Clostridia | Clostridiales | Lachnospiraceae | *unclassified* | 1.15 | 0.75 | Connectors |
| ZOTU_11245 | Pseudomonadota | Alphaproteobacteria | Rhizobiales | Bradyrhizobiaceae | *unclassified* | 0.77 | 0.76 | Connectors |
| ZOTU_11832 | Pseudomonadota | Gammaproteobacteria | Pseudomonadales | Pseudomonadaceae | *Pseudomonas* | 0.94 | 0.76 | Connectors |
| ZOTU_78 | Pseudomonadota | Betaproteobacteria | Burkholderiales | Comamonadaceae | *Curvibacter* | 0.77 | 0.76 | Connectors |
| ZOTU_10622 | Pseudomonadota | Betaproteobacteria | Burkholderiales | Comamonadaceae | *unclassified* | -0.64 | 0.78 | Connectors |
| ZOTU_12756 | Pseudomonadota | Betaproteobacteria | Burkholderiales | Comamonadaceae | *unclassified* | -0.08 | 0.78 | Connectors |
| ZOTU_129 | Pseudomonadota | Betaproteobacteria | Burkholderiales | Burkholderiaceae | *Paraburkholderia* | -0.40 | 0.78 | Connectors |
| ZOTU_13531 | Pseudomonadota | Alphaproteobacteria | Rhizobiales | Bradyrhizobiaceae | *unclassified* | -0.28 | 0.78 | Connectors |
| ZOTU_14695 | Actinomycetota | Actinomycetota | Mycobacteriales | Mycobacteriaceae | *Mycobacterium* | 0.18 | 0.78 | Connectors |
| ZOTU_14710 | Pseudomonadota | Betaproteobacteria | Burkholderiales | Comamonadaceae | *unclassified* | -0.28 | 0.78 | Connectors |
| ZOTU_154 | Pseudomonadota | Alphaproteobacteria | unclassified | SAR11 | *Candidatus_Pelagibacter* | 0.00 | 0.78 | Connectors |
| ZOTU_3381 | Pseudomonadota | unclassified | unclassified | unclassified | *unclassified* | -0.28 | 0.78 | Connectors |
| ZOTU_3917 | Pseudomonadota | Betaproteobacteria | Burkholderiales | unclassified | *unclassified* | 0.00 | 0.78 | Connectors |
| ZOTU_454 | Pseudomonadota | Betaproteobacteria | Nitrosomonadales | unclassified | *unclassified* | 0.00 | 0.78 | Connectors |
| ZOTU_6357 | Pseudomonadota | unclassified | unclassified | unclassified | *unclassified* | 0.00 | 0.78 | Connectors |
| ZOTU_6609 | Pseudomonadota | unclassified | unclassified | unclassified | *unclassified* | -0.28 | 0.78 | Connectors |
| ZOTU_11175 | Actinomycetota | Actinomycetota | Mycobacteriales | unclassified | *unclassified* | 0.32 | 0.81 | Connectors |
| ZOTU_11042 | Pseudomonadota | Betaproteobacteria | Burkholderiales | Comamonadaceae | *unclassified* | -0.08 | 0.81 | Connectors |
| ZOTU_12115 | Pseudomonadota | Gammaproteobacteria | Pseudomonadales | Pseudomonadaceae | *Pseudomonas* | -0.08 | 0.81 | Connectors |
| ZOTU_47 | Pseudomonadota | Alphaproteobacteria | unclassified | SAR11 | *Candidatus_Pelagibacter* | 0.00 | 0.81 | Connectors |
| ZOTU_7830 | Pseudomonadota | Betaproteobacteria | Burkholderiales | unclassified | *unclassified* | -0.08 | 0.81 | Connectors |
| ZOTU_201 | Pseudomonadota | Betaproteobacteria | Burkholderiales | Comamonadaceae | *unclassified* | 0.00 | 0.84 | Connectors |
| ZOTU_650 | Bacillota | Clostridia | Clostridiales | Lachnospiraceae | *unclassified* | 2.65 | 0.27 | Module hubs |
| ZOTU_6467 | Pseudomonadota | Alphaproteobacteria | Sphingomonadales | Sphingomonadaceae | *Sphingomonas* | 2.73 | 0.36 | Module hubs |
| ZOTU_5982 | Pseudomonadota | Gammaproteobacteria | Pseudomonadales | Moraxellaceae | *Acinetobacter* | 2.55 | 0.60 | Module hubs |
| ZOTU_14 | Ascomycota | unclassfied | unclassfied | unclassfied | unclassfied | -1.00 | 0.75 | Connectors |
| ZOTU_155 | unclassfied | unclassfied | unclassfied | unclassfied | unclassfied | 0.00 | 0.75 | Connectors |
| ZOTU_225 | Ascomycota | Dothideomycetes | Capnodiales | unclassfied | unclassfied | 0.00 | 0.75 | Connectors |
| ZOTU_56 | Ascomycota | Dothideomycetes | Dothideales | Aureobasidiaceae | Aureobasidium | -1.81 | 0.68 | Connectors |
| ZOTU_69 | Basidiomycota | Tremellomycetes | unclassfied | unclassfied | unclassfied | 0.00 | 0.75 | Connectors |

**TABLE S3** Detailed taxonomic information for module hubs and connectors of wood bacterial and fungal network.

| **Nodes-id** | **Phylum** | **Class** | **Order** | **Family** | **Genus** | **Zi** | **Pi** | **Type** |
| --- | --- | --- | --- | --- | --- | --- | --- | --- |
| ZOTU_10035 | Pseudomonadota | Gammaproteobacteria | Pseudomonadales | Moraxellaceae | Acinetobacter | -0.96 | 0.78 | Connectors |
| ZOTU_10046 | Pseudomonadota | unclassified | unclassified | unclassified | unclassified | -0.80 | 0.75 | Connectors |
| ZOTU_101 | Pseudomonadota | Alphaproteobacteria | Sphingomonadales | Erythrobacteraceae | Novosphingobium | -1.51 | 0.75 | Connectors |
| ZOTU_10345 | Pseudomonadota | Gammaproteobacteria | Xanthomonadales | Rhodanobacteraceae | unclassified | 0.31 | 0.76 | Connectors |
| ZOTU_10614 | Pseudomonadota | Betaproteobacteria | Burkholderiales | Burkholderiaceae | unclassified | -0.96 | 0.75 | Connectors |
| ZOTU_10710 | Pseudomonadota | Gammaproteobacteria | Pseudomonadales | Moraxellaceae | Acinetobacter | -0.41 | 0.67 | Connectors |
| ZOTU_108 | Bacteroidota | Flavobacteriia | Flavobacteriales | Weeksellaceae | Chryseobacterium | -0.96 | 0.75 | Connectors |
| ZOTU_10876 | Pseudomonadota | Gammaproteobacteria | Pseudomonadales | Moraxellaceae | unclassified | 0.87 | 0.75 | Connectors |
| ZOTU_1104 | Acidobacteria | Acidobacteria_Gp1 | unclassified | unclassified | Granulicella | -0.96 | 0.75 | Connectors |
| ZOTU_11057 | Pseudomonadota | Gammaproteobacteria | unclassified | unclassified | unclassified | -0.96 | 0.75 | Connectors |
| ZOTU_11141 | Actinomycetota | Actinomycetota | unclassified | unclassified | unclassified | -0.82 | 0.69 | Connectors |
| ZOTU_11226 | Pseudomonadota | Gammaproteobacteria | unclassified | unclassified | unclassified | 1.33 | 0.70 | Connectors |
| ZOTU_11315 | Pseudomonadota | Gammaproteobacteria | Enterobacterales | unclassified | unclassified | -0.71 | 0.75 | Connectors |
| ZOTU_11327 | Pseudomonadota | unclassified | unclassified | unclassified | unclassified | -0.96 | 0.75 | Connectors |
| ZOTU_1158 | Pseudomonadota | Alphaproteobacteria | Sphingomonadales | Erythrobacteraceae | Novosphingobium | -0.90 | 0.75 | Connectors |
| ZOTU_11610 | Pseudomonadota | Gammaproteobacteria | unclassified | unclassified | unclassified | 0.00 | 0.75 | Connectors |
| ZOTU_11874 | Pseudomonadota | Gammaproteobacteria | Pseudomonadales | Pseudomonadaceae | Pseudomonas | -1.51 | 0.75 | Connectors |
| ZOTU_1193 | Acidobacteria | Acidobacteria_Gp1 | unclassified | unclassified | Granulicella | -0.90 | 0.75 | Connectors |
| ZOTU_12052 | Pseudomonadota | Gammaproteobacteria | Pseudomonadales | Moraxellaceae | unclassified | 0.00 | 0.75 | Connectors |
| ZOTU_12345 | Pseudomonadota | Gammaproteobacteria | Pseudomonadales | Pseudomonadaceae | Pseudomonas | -0.96 | 0.78 | Connectors |
| ZOTU_12693 | Bacteroidota | Sphingobacteriia | Sphingobacteriales | Sphingobacteriaceae | Mucilaginibacter | 0.37 | 0.62 | Connectors |
| ZOTU_12721 | Pseudomonadota | Gammaproteobacteria | Pseudomonadales | unclassified | unclassified | -0.55 | 0.75 | Connectors |
| ZOTU_12750 | unclassified | unclassified | unclassified | unclassified | unclassified | -0.96 | 0.75 | Connectors |
| ZOTU_128 | Bacteroidota | Chitinophagia | Chitinophagales | Chitinophagaceae | Taibaiella | -0.80 | 0.81 | Connectors |
| ZOTU_12800 | unclassified | unclassified | unclassified | unclassified | unclassified | -0.96 | 0.75 | Connectors |
| ZOTU_131 | Pseudomonadota | Alphaproteobacteria | Rhodospirillales | Acetobacteraceae | Acidisoma | -0.90 | 0.78 | Connectors |
| ZOTU_13504 | Pseudomonadota | Alphaproteobacteria | Sphingomonadales | Sphingomonadaceae | Sphingomonas | -0.55 | 0.75 | Connectors |
| ZOTU_13733 | Pseudomonadota | Gammaproteobacteria | Xanthomonadales | Rhodanobacteraceae | Dyella | -0.55 | 0.76 | Connectors |
| ZOTU_13796 | Pseudomonadota | unclassified | unclassified | unclassified | unclassified | -1.51 | 0.75 | Connectors |
| ZOTU_13914 | Pseudomonadota | Betaproteobacteria | Burkholderiales | unclassified | unclassified | -0.71 | 0.75 | Connectors |
| ZOTU_141 | Bacteroidota | Cytophagia | Cytophagales | Cytophagaceae | Dyadobacter | 1.82 | 0.69 | Connectors |
| ZOTU_14241 | Pseudomonadota | unclassified | unclassified | unclassified | unclassified | -1.45 | 0.75 | Connectors |
| ZOTU_14285 | Pseudomonadota | Betaproteobacteria | unclassified | unclassified | unclassified | -0.96 | 0.75 | Connectors |
| ZOTU_145 | Verrucomicrobia | Verrucomicrobiae | Verrucomicrobiales | Verrucomicrobiaceae | Luteolibacter | -0.96 | 0.69 | Connectors |
| ZOTU_14547 | Pseudomonadota | Gammaproteobacteria | Enterobacterales | unclassified | unclassified | 0.82 | 0.63 | Connectors |
| ZOTU_14659 | Pseudomonadota | Gammaproteobacteria | Xanthomonadales | Rhodanobacteraceae | unclassified | -0.96 | 0.75 | Connectors |
| ZOTU_152 | Bacteroidota | Chitinophagia | Chitinophagales | Chitinophagaceae | Taibaiella | -0.65 | 0.76 | Connectors |
| ZOTU_160 | Pseudomonadota | Gammaproteobacteria | Pseudomonadales | Pseudomonadaceae | Pseudomonas | -0.30 | 0.75 | Connectors |
| ZOTU_166 | Acidobacteria | Acidobacteria_Gp1 | unclassified | unclassified | Terriglobus | -0.14 | 0.71 | Connectors |
| ZOTU_1733 | Pseudomonadota | Gammaproteobacteria | unclassified | unclassified | unclassified | -0.20 | 0.76 | Connectors |
| ZOTU_177 | Pseudomonadota | Alphaproteobacteria | Sphingomonadales | Erythrobacteraceae | Novosphingobium | -0.96 | 0.75 | Connectors |
| ZOTU_1799 | Acidobacteria | Acidobacteria_Gp1 | unclassified | unclassified | unclassified | -0.65 | 0.69 | Connectors |
| ZOTU_1948 | Pseudomonadota | Betaproteobacteria | Burkholderiales | unclassified | unclassified | -0.96 | 0.69 | Connectors |
| ZOTU_220 | Pseudomonadota | Alphaproteobacteria | Sphingomonadales | unclassified | unclassified | -0.90 | 0.78 | Connectors |
| ZOTU_228 | unclassified | unclassified | unclassified | unclassified | unclassified | -0.96 | 0.75 | Connectors |
| ZOTU_232 | Acidobacteria | Acidobacteria_Gp1 | unclassified | unclassified | Edaphobacter | -1.16 | 0.75 | Connectors |
| ZOTU_2336 | Pseudomonadota | Gammaproteobacteria | unclassified | unclassified | unclassified | 0.00 | 0.75 | Connectors |
| ZOTU_235 | Pseudomonadota | Gammaproteobacteria | Pseudomonadales | Pseudomonadaceae | Pseudomonas | -0.97 | 0.78 | Connectors |
| ZOTU_236 | unclassified | unclassified | unclassified | unclassified | unclassified | -0.96 | 0.75 | Connectors |
| ZOTU_24 | Pseudomonadota | Gammaproteobacteria | Enterobacterales | unclassified | unclassified | -0.67 | 0.64 | Connectors |
| ZOTU_26 | Pseudomonadota | Gammaproteobacteria | Enterobacterales | Enterobacteriaceae | unclassified | -0.71 | 0.75 | Connectors |
| ZOTU_263 | Bacteroidota | Flavobacteriia | Flavobacteriales | Flavobacteriaceae | Flavobacterium | -0.90 | 0.75 | Connectors |
| ZOTU_295 | Acidobacteria | Acidobacteria_Gp1 | unclassified | unclassified | Granulicella | -0.96 | 0.75 | Connectors |
| ZOTU_30 | Pseudomonadota | Gammaproteobacteria | Xanthomonadales | Rhodanobacteraceae | Dyella | 1.15 | 0.75 | Connectors |
| ZOTU_3119 | Pseudomonadota | Gammaproteobacteria | Xanthomonadales | Rhodanobacteraceae | unclassified | -0.71 | 0.78 | Connectors |
| ZOTU_312 | Bacteroidota | Sphingobacteriia | Sphingobacteriales | Sphingobacteriaceae | Mucilaginibacter | 1.14 | 0.66 | Connectors |
| ZOTU_332 | Bacteroidota | unclassified | unclassified | unclassified | unclassified | 0.00 | 0.78 | Connectors |
| ZOTU_3320 | Pseudomonadota | Gammaproteobacteria | unclassified | unclassified | unclassified | -0.55 | 0.75 | Connectors |
| ZOTU_3653 | Pseudomonadota | Betaproteobacteria | Burkholderiales | Burkholderiaceae | unclassified | -0.96 | 0.75 | Connectors |
| ZOTU_3909 | Pseudomonadota | Gammaproteobacteria | unclassified | unclassified | unclassified | -0.58 | 0.75 | Connectors |
| ZOTU_403 | Pseudomonadota | Alphaproteobacteria | Rhizobiales | Brucellaceae | unclassified | -0.80 | 0.76 | Connectors |
| ZOTU_415 | Pseudomonadota | Alphaproteobacteria | Sphingomonadales | Sphingomonadaceae | unclassified | -0.82 | 0.69 | Connectors |
| ZOTU_4254 | Pseudomonadota | Gammaproteobacteria | Pseudomonadales | Moraxellaceae | Acinetobacter | 0.87 | 0.75 | Connectors |
| ZOTU_436 | Acidobacteria | Acidobacteria_Gp1 | unclassified | unclassified | unclassified | -0.80 | 0.75 | Connectors |
| ZOTU_45 | Actinomycetota | Actinomycetota | Micrococcales | Microbacteriaceae | Curtobacterium | -0.96 | 0.75 | Connectors |
| ZOTU_4781 | Pseudomonadota | Gammaproteobacteria | unclassified | unclassified | unclassified | -0.71 | 0.75 | Connectors |
| ZOTU_49 | Pseudomonadota | Alphaproteobacteria | Sphingomonadales | Sphingomonadaceae | Sphingomonas | -0.67 | 0.64 | Connectors |
| ZOTU_4961 | Pseudomonadota | Gammaproteobacteria | Pseudomonadales | Moraxellaceae | Acinetobacter | -1.27 | 0.63 | Connectors |
| ZOTU_5133 | Pseudomonadota | Gammaproteobacteria | unclassified | unclassified | unclassified | -0.20 | 0.69 | Connectors |
| ZOTU_550 | Pseudomonadota | Betaproteobacteria | Burkholderiales | Alcaligenaceae | unclassified | -0.90 | 0.78 | Connectors |
| ZOTU_554 | unclassified | unclassified | unclassified | unclassified | unclassified | -0.96 | 0.75 | Connectors |
| ZOTU_571 | Verrucomicrobia | Verrucomicrobiae | Verrucomicrobiales | Verrucomicrobiaceae | Luteolibacter | -0.39 | 0.64 | Connectors |
| ZOTU_5869 | Pseudomonadota | Alphaproteobacteria | Sphingomonadales | Erythrobacteraceae | Novosphingobium | -0.30 | 0.75 | Connectors |
| ZOTU_6163 | Pseudomonadota | Alphaproteobacteria | Sphingomonadales | Erythrobacteraceae | Novosphingobium | -0.30 | 0.75 | Connectors |
| ZOTU_617 | Pseudomonadota | Alphaproteobacteria | Sphingomonadales | unclassified | unclassified | -0.30 | 0.75 | Connectors |
| ZOTU_632 | Bacteroidota | Flavobacteriia | Flavobacteriales | Flavobacteriaceae | Flavobacterium | -0.96 | 0.69 | Connectors |
| ZOTU_64 | Bacteroidota | Sphingobacteriia | Sphingobacteriales | Sphingobacteriaceae | unclassified | 0.62 | 0.67 | Connectors |
| ZOTU_6440 | Pseudomonadota | Betaproteobacteria | Burkholderiales | unclassified | unclassified | -0.71 | 0.75 | Connectors |
| ZOTU_653 | Verrucomicrobia | Verrucomicrobiae | Verrucomicrobiales | Verrucomicrobiaceae | Luteolibacter | -0.65 | 0.69 | Connectors |
| ZOTU_657 | Verrucomicrobia | Verrucomicrobiae | Verrucomicrobiales | Verrucomicrobiaceae | Luteolibacter | -0.96 | 0.75 | Connectors |
| ZOTU_6672 | Pseudomonadota | unclassified | unclassified | unclassified | unclassified | -0.96 | 0.75 | Connectors |
| ZOTU_67 | Pseudomonadota | Gammaproteobacteria | Enterobacterales | unclassified | unclassified | -0.82 | 0.75 | Connectors |
| ZOTU_6700 | Pseudomonadota | Gammaproteobacteria | Xanthomonadales | Rhodanobacteraceae | unclassified | -0.83 | 0.69 | Connectors |
| ZOTU_6723 | Pseudomonadota | Gammaproteobacteria | Pseudomonadales | unclassified | unclassified | -0.96 | 0.75 | Connectors |
| ZOTU_6842 | Pseudomonadota | Gammaproteobacteria | Enterobacterales | unclassified | unclassified | -0.20 | 0.69 | Connectors |
| ZOTU_689 | Actinomycetota | Actinomycetota | Micrococcales | Microbacteriaceae | unclassified | -0.96 | 0.75 | Connectors |
| ZOTU_69 | unclassified | unclassified | unclassified | unclassified | unclassified | 0.00 | 0.75 | Connectors |
| ZOTU_692 | Pseudomonadota | Betaproteobacteria | Burkholderiales | Comamonadaceae | Ramlibacter | -0.30 | 0.75 | Connectors |
| ZOTU_696 | Pseudomonadota | Betaproteobacteria | Burkholderiales | Alcaligenaceae | unclassified | -0.80 | 0.69 | Connectors |
| ZOTU_698 | Bacteroidota | Flavobacteriia | Flavobacteriales | Flavobacteriaceae | Flavobacterium | 0.67 | 0.76 | Connectors |
| ZOTU_7293 | Pseudomonadota | Gammaproteobacteria | unclassified | unclassified | unclassified | -0.41 | 0.68 | Connectors |
| ZOTU_7441 | Pseudomonadota | unclassified | unclassified | unclassified | unclassified | -0.96 | 0.75 | Connectors |
| ZOTU_7457 | Pseudomonadota | Betaproteobacteria | Burkholderiales | Burkholderiaceae | unclassified | -1.27 | 0.75 | Connectors |
| ZOTU_746 | Pseudomonadota | Alphaproteobacteria | Sphingomonadales | Erythrobacteraceae | Novosphingobium | -1.16 | 0.75 | Connectors |
| ZOTU_7800 | unclassified | unclassified | unclassified | unclassified | unclassified | -1.51 | 0.75 | Connectors |
| ZOTU_783 | Bacteroidota | Flavobacteriia | Flavobacteriales | Flavobacteriaceae | Flavobacterium | -0.30 | 0.78 | Connectors |
| ZOTU_7947 | Pseudomonadota | Gammaproteobacteria | unclassified | unclassified | unclassified | 1.15 | 0.75 | Connectors |
| ZOTU_8 | Pseudomonadota | Gammaproteobacteria | Xanthomonadales | Rhodanobacteraceae | unclassified | 0.06 | 0.67 | Connectors |
| ZOTU_8160 | Pseudomonadota | Betaproteobacteria | Burkholderiales | Burkholderiaceae | unclassified | -0.71 | 0.78 | Connectors |
| ZOTU_82 | Pseudomonadota | Betaproteobacteria | Burkholderiales | Burkholderiaceae | Paraburkholderia | 1.15 | 0.75 | Connectors |
| ZOTU_822 | Pseudomonadota | Alphaproteobacteria | Sphingomonadales | Erythrobacteraceae | Novosphingobium | 0.00 | 0.75 | Connectors |
| ZOTU_8264 | Pseudomonadota | Alphaproteobacteria | Sphingomonadales | Erythrobacteraceae | Novosphingobium | -0.30 | 0.75 | Connectors |
| ZOTU_857 | Thaumarchaeota | unclassified | Nitrososphaerales | Nitrososphaeraceae | Nitrososphaera | -0.96 | 0.75 | Connectors |
| ZOTU_86 | Pseudomonadota | Betaproteobacteria | Burkholderiales | unclassified | unclassified | -0.67 | 0.73 | Connectors |
| ZOTU_8645 | Pseudomonadota | Betaproteobacteria | Burkholderiales | Burkholderiaceae | unclassified | -0.96 | 0.75 | Connectors |
| ZOTU_88 | Verrucomicrobia | Verrucomicrobiae | Verrucomicrobiales | Verrucomicrobiaceae | Luteolibacter | -0.96 | 0.75 | Connectors |
| ZOTU_9105 | Pseudomonadota | unclassified | unclassified | unclassified | unclassified | 1.15 | 0.81 | Connectors |
| ZOTU_9162 | Pseudomonadota | Alphaproteobacteria | Sphingomonadales | Sphingomonadaceae | Sphingomonas | -0.55 | 0.78 | Connectors |
| ZOTU_925 | Chlamydiae | Chlamydiia | Chlamydiales | unclassified | unclassified | 1.15 | 0.75 | Connectors |
| ZOTU_9266 | Pseudomonadota | Betaproteobacteria | Burkholderiales | Comamonadaceae | unclassified | -0.71 | 0.75 | Connectors |
| ZOTU_9309 | Pseudomonadota | Gammaproteobacteria | unclassified | unclassified | unclassified | -0.96 | 0.75 | Connectors |
| ZOTU_9521 | Pseudomonadota | Gammaproteobacteria | Pseudomonadales | Moraxellaceae | Acinetobacter | -0.96 | 0.78 | Connectors |
| ZOTU_9535 | Pseudomonadota | Gammaproteobacteria | Xanthomonadales | unclassified | unclassified | -0.71 | 0.75 | Connectors |
| ZOTU_956 | Bacteroidota | Chitinophagia | Chitinophagales | Chitinophagaceae | unclassified | -0.96 | 0.75 | Connectors |
| ZOTU_960 | Pseudomonadota | Gammaproteobacteria | Pseudomonadales | Pseudomonadaceae | Pseudomonas | -0.58 | 0.69 | Connectors |
| ZOTU_971 | Bacteroidota | Chitinophagia | Chitinophagales | Chitinophagaceae | Chitinophaga | -0.38 | 0.65 | Connectors |
| ZOTU_14264 | Pseudomonadota | Betaproteobacteria | Burkholderiales | Burkholderiaceae | unclassified | 4.16 | 0.15 | Module hubs |
| ZOTU_207 | Pseudomonadota | Betaproteobacteria | Burkholderiales | Oxalobacteraceae | Janthinobacterium | 2.61 | 0.36 | Module hubs |
| ZOTU_2358 | Pseudomonadota | Betaproteobacteria | Burkholderiales | Comamonadaceae | unclassified | 5.34 | 0.24 | Module hubs |
| ZOTU_243 | Bacteroidota | Chitinophagia | Chitinophagales | Chitinophagaceae | Chitinophaga | 2.70 | 0.42 | Module hubs |
| ZOTU_3238 | Pseudomonadota | Gammaproteobacteria | Pseudomonadales | Moraxellaceae | Acinetobacter | 2.84 | 0.53 | Module hubs |
| ZOTU_6 | Pseudomonadota | Gammaproteobacteria | Xanthomonadales | Xanthomonadaceae | Pseudoxanthomonas | 2.85 | 0.21 | Module hubs |
| ZOTU_110 | Basidiomycota | Agaricostilbomycetes | Agaricostilbales | Jianyuniaceae | Jianyunia | -1.06 | 0.69 | Connectors |
| ZOTU_1180 | Ascomycota | Saccharomycetes | Saccharomycetales | Pichiaceae | Nakazawaea | 0.00 | 0.75 | Connectors |
| ZOTU_123 | Ascomycota | unclassfied | unclassfied | unclassfied | unclassfied | -1.08 | 0.69 | Connectors |
| ZOTU_1326 | Ascomycota | unclassfied | unclassfied | unclassfied | unclassfied | -1.63 | 0.68 | Connectors |
| ZOTU_1327 | Ascomycota | Saccharomycetes | Saccharomycetales | Pichiaceae | Nakazawaea | -1.06 | 0.68 | Connectors |
| ZOTU_135 | Ascomycota | Saccharomycetes | Saccharomycetales | Debaryomycetaceae | Yamadazyma | -1.43 | 0.75 | Connectors |
| ZOTU_167 | Ascomycota | Sordariomycetes | Hypocreales | Nectriaceae | Xenoacremonium | -1.10 | 0.68 | Connectors |
| ZOTU_2 | Ascomycota | Dothideomycetes | Botryosphaeriales | Botryosphaeriaceae | Diplodia | -1.55 | 0.75 | Connectors |
| ZOTU_214 | Ascomycota | unclassfied | unclassfied | unclassfied | unclassfied | -1.25 | 0.63 | Connectors |
| ZOTU_25 | unclassfied | unclassfied | unclassfied | unclassfied | unclassfied | -1.43 | 0.75 | Connectors |
| ZOTU_3201 | unclassfied | unclassfied | unclassfied | unclassfied | unclassfied | -1.43 | 0.75 | Connectors |
| ZOTU_347 | Basidiomycota | Microbotryomycetes | unclassfied | unclassfied | unclassfied | -1.43 | 0.75 | Connectors |
| ZOTU_357 | Ascomycota | Sordariomycetes | Hypocreales | Hypocreaceae | unclassfied | -1.43 | 0.78 | Connectors |
| ZOTU_4 | Ascomycota | Saccharomycetes | Saccharomycetales | Pichiaceae | Nakazawaea | -1.63 | 0.69 | Connectors |
| ZOTU_464 | Mortierellomycota | Mortierellomycetes | Mortierellales | Mortierellaceae | Mortierella | -1.24 | 0.78 | Connectors |
| ZOTU_5 | Ascomycota | Sordariomycetes | Ophiostomatales | Ophiostomataceae | Leptographium | 0.78 | 0.66 | Connectors |
| ZOTU_50 | Ascomycota | Eurotiomycetes | Chaetothyriales | Herpotrichiellaceae | unclassfied | -0.04 | 0.63 | Connectors |
| ZOTU_56 | Ascomycota | Dothideomycetes | Dothideales | Aureobasidiaceae | Aureobasidium | -1.24 | 0.75 | Connectors |
| ZOTU_58 | Ascomycota | Sordariomycetes | Ophiostomatales | Ophiostomataceae | Ceratocystiopsis | -1.25 | 0.73 | Connectors |
| ZOTU_614 | Ascomycota | Sordariomycetes | unclassfied | unclassfied | unclassfied | -1.55 | 0.78 | Connectors |
| ZOTU_62 | Ascomycota | Sordariomycetes | Hypocreales | Nectriaceae | Xenoacremonium | -1.24 | 0.75 | Connectors |
| ZOTU_63 | Ascomycota | Eurotiomycetes | Phaeomoniellales | Phaeomoniellaceae | Aequabiliella | -0.69 | 0.67 | Connectors |
| ZOTU_90 | unclassfied | unclassfied | unclassfied | unclassfied | unclassfied | -1.24 | 0.75 | Connectors |

**TABLE S4** Detailed bacterial taxonomic information for enriched taxa of pine wood nematode and infested wood.

| **Group** | **Vegetation type** | **ZOTU** | **phylum** | **class** | **order** | **family** | **genus** | **logFC** | **logCPM** | **PValue** | **FDR** |
| --- | --- | --- | --- | --- | --- | --- | --- | --- | --- | --- | --- |
| Nematode | CEBLF | ZOTU_22 | Proteobacteria | Gammaproteobacteria | Pseudomonadales | Pseudomonadaceae | Pseudomonas | 2.01 | 13.10 | 1.60E-11 | 1.36E-09 |
|  |  | ZOTU_35 | Proteobacteria | Betaproteobacteria | Burkholderiales | Comamonadaceae | unclassified | 1.95 | 11.94 | 1.20E-10 | 8.74E-09 |
|  |  | ZOTU_37 | Proteobacteria | Gammaproteobacteria | Xanthomonadales | Xanthomonadaceae | Stenotrophomonas | 1.50 | 11.54 | 1.66E-08 | 7.99E-07 |
|  |  | ZOTU_20 | Proteobacteria | Betaproteobacteria | Burkholderiales | Burkholderiaceae | Ralstonia | 1.77 | 12.98 | 5.21E-07 | 1.79E-05 |
|  |  | ZOTU_44 | Proteobacteria | Gammaproteobacteria | Xanthomonadales | Xanthomonadaceae | Stenotrophomonas | 1.21 | 11.47 | 3.27E-05 | 6.69E-04 |
|  | TCBF | ZOTU_35 | Proteobacteria | Betaproteobacteria | Burkholderiales | Comamonadaceae | unclassified | 2.33 | 11.85 | 2.55E-06 | 3.92E-05 |
|  |  | ZOTU_37 | Proteobacteria | Gammaproteobacteria | Xanthomonadales | Xanthomonadaceae | Stenotrophomonas | 1.84 | 11.46 | 2.90E-05 | 3.95E-04 |
|  |  | ZOTU_20 | Proteobacteria | Betaproteobacteria | Burkholderiales | Burkholderiaceae | Ralstonia | 2.16 | 12.89 | 1.60E-04 | 1.93E-03 |
|  |  | ZOTU_22 | Proteobacteria | Gammaproteobacteria | Pseudomonadales | Pseudomonadaceae | Pseudomonas | 1.81 | 12.96 | 7.06E-04 | 7.39E-03 |
|  |  | ZOTU_33 | Proteobacteria | Gammaproteobacteria | Pseudomonadales | Moraxellaceae | Acinetobacter | 1.64 | 11.74 | 3.25E-03 | 3.05E-02 |
|  |  | ZOTU_27 | Proteobacteria | Betaproteobacteria | Burkholderiales | Burkholderiaceae | Ralstonia | 1.48 | 10.74 | 3.54E-03 | 3.30E-02 |
|  |  | ZOTU_44 | Proteobacteria | Gammaproteobacteria | Xanthomonadales | Xanthomonadaceae | Stenotrophomonas | 1.43 | 11.44 | 3.65E-03 | 3.40E-02 |
| Wood | CEBLF | ZOTU_65 | Proteobacteria | Gammaproteobacteria | Xanthomonadales | Rhodanobacteraceae | Luteibacter | 3.80 | 11.44 | 1.14E+01 | 3.60E-15 |
|  |  | ZOTU_331 | Proteobacteria | Alphaproteobacteria | Sphingomonadales | Sphingomonadaceae | Sphingomonas | 2.95 | 8.91 | 8.91E+00 | 1.01E-13 |
|  |  | ZOTU_13886 | Proteobacteria | Gammaproteobacteria | unclassified | unclassified | unclassified | 2.52 | 9.61 | 9.61E+00 | 1.23E-07 |
|  |  | ZOTU_9 | Proteobacteria | Betaproteobacteria | Burkholderiales | Comamonadaceae | unclassified | 2.38 | 10.98 | 1.10E+01 | 4.62E-05 |
|  |  | ZOTU_26 | Proteobacteria | Gammaproteobacteria | Enterobacterales | Enterobacteriaceae | unclassified | 2.42 | 12.08 | 1.21E+01 | 8.42E-05 |
|  |  | ZOTU_9304 | Proteobacteria | Gammaproteobacteria | Pseudomonadales | Moraxellaceae | Acinetobacter | 1.66 | 10.89 | 1.09E+01 | 2.72E-04 |
|  |  | ZOTU_4254 | Proteobacteria | Gammaproteobacteria | Pseudomonadales | Moraxellaceae | Acinetobacter | 1.66 | 8.98 | 8.98E+00 | 4.56E-04 |
|  |  | ZOTU_13126 | Proteobacteria | Gammaproteobacteria | Pseudomonadales | unclassified | unclassified | 1.75 | 9.08 | 9.08E+00 | 5.00E-04 |
|  |  | ZOTU_81 | Proteobacteria | Alphaproteobacteria | Sphingomonadales | Sphingomonadaceae | Sphingomonas | 1.46 | 9.12 | 9.12E+00 | 6.63E-04 |
|  |  | ZOTU_83 | Proteobacteria | Betaproteobacteria | Burkholderiales | Burkholderiaceae | unclassified | 1.59 | 10.89 | 1.09E+01 | 1.41E-03 |
|  |  | ZOTU_5731 | Proteobacteria | Gammaproteobacteria | Pseudomonadales | Moraxellaceae | Acinetobacter | 1.58 | 9.18 | 9.18E+00 | 1.63E-03 |
|  |  | ZOTU_9374 | Proteobacteria | Gammaproteobacteria | Pseudomonadales | Moraxellaceae | unclassified | 1.43 | 7.88 | 7.88E+00 | 1.69E-03 |
|  |  | ZOTU_4 | Proteobacteria | Gammaproteobacteria | Pseudomonadales | Moraxellaceae | Acinetobacter | 1.74 | 16.32 | 1.63E+01 | 1.87E-03 |
|  | NEDBF | ZOTU_5 | Proteobacteria | Gammaproteobacteria | Enterobacterales | Yersiniaceae | unclassified | 7.50 | 14.38 | 1.44E+01 | 9.91E-05 |
|  |  | ZOTU_89 | Proteobacteria | Betaproteobacteria | Burkholderiales | Burkholderiaceae | Paraburkholderia | 3.43 | 10.36 | 1.04E+01 | 1.02E-03 |
|  | TCBF | ZOTU_65 | Proteobacteria | Gammaproteobacteria | Xanthomonadales | Rhodanobacteraceae | Luteibacter | 4.76 | 11.64 | 1.16E+01 | 8.90E-19 |
|  |  | ZOTU_9374 | Proteobacteria | Gammaproteobacteria | Pseudomonadales | Moraxellaceae | unclassified | 2.35 | 7.88 | 7.88E+00 | 4.48E-06 |
|  |  | ZOTU_9 | Proteobacteria | Betaproteobacteria | Burkholderiales | Comamonadaceae | unclassified | 2.92 | 11.02 | 1.10E+01 | 5.52E-05 |
|  |  | ZOTU_9304 | Proteobacteria | Gammaproteobacteria | Pseudomonadales | Moraxellaceae | Acinetobacter | 2.25 | 10.90 | 1.09E+01 | 6.98E-05 |
|  |  | ZOTU_13126 | Proteobacteria | Gammaproteobacteria | Pseudomonadales | unclassified | unclassified | 2.41 | 9.09 | 9.09E+00 | 8.29E-05 |
|  |  | ZOTU_4 | Proteobacteria | Gammaproteobacteria | Pseudomonadales | Moraxellaceae | Acinetobacter | 2.53 | 16.30 | 1.63E+01 | 1.45E-04 |
|  |  | ZOTU_14270 | Proteobacteria | Gammaproteobacteria | Pseudomonadales | Pseudomonadaceae | unclassified | 2.00 | 8.12 | 8.12E+00 | 7.63E-04 |
|  |  | ZOTU_5731 | Proteobacteria | Gammaproteobacteria | Pseudomonadales | Moraxellaceae | Acinetobacter | 2.12 | 9.19 | 9.19E+00 | 7.66E-04 |
|  |  | ZOTU_13886 | Proteobacteria | Gammaproteobacteria | unclassified | unclassified | unclassified | 2.15 | 9.47 | 9.47E+00 | 1.65E-03 |

**Note:** TCBF means temperate coniferous-broadleaf mixed forest, NEDBF means northern subtropical evergreen-deciduous broadleaf mixed forest, CEBLF means central subtropical evergreen broad-leaved forests.

**TABLE S****5** The count and proportion of core taxa associated with pine wood nematode and infest wood at the phylum, class, order, and family level.

| **Group** | **Classification** | **Core taxa** | **Count** | **Proportion** |
| --- | --- | --- | --- | --- |
| Nematode | Phylum | Proteobacteria | 55 | 59.78% |
|  |  | Firmicutes | 12 | 13.04% |
|  |  | Bacteroidetes | 11 | 11.96% |
|  |  | Actinobacteria | 9 | 9.78% |
|  |  | unclassified | 3 | 3.26% |
|  |  | Ignavibacteriae | 1 | 1.09% |
|  |  | Euryarchaeota | 1 | 1.09% |
|  | Class | Betaproteobacteria | 29 | 31.52% |
|  |  | Gammaproteobacteria | 12 | 13.04% |
|  |  | Actinobacteria | 9 | 9.78% |
|  |  | unclassified | 9 | 9.78% |
|  |  | Alphaproteobacteria | 9 | 9.78% |
|  |  | Clostridia | 8 | 8.70% |
|  |  | Bacteroidia | 6 | 6.52% |
|  |  | Flavobacteriia | 4 | 4.35% |
|  |  | Bacilli | 3 | 3.26% |
|  |  | Erysipelotrichia | 1 | 1.09% |
|  |  | Ignavibacteria | 1 | 1.09% |
|  |  | Methanobacteria | 1 | 1.09% |
|  | Order | Burkholderiales | 26 | 28.26% |
|  |  | unclassified | 16 | 17.39% |
|  |  | Mycobacteriales | 9 | 9.78% |
|  |  | Pseudomonadales | 8 | 8.70% |
|  |  | Clostridiales | 8 | 8.70% |
|  |  | Bacteroidales | 6 | 6.52% |
|  |  | Flavobacteriales | 4 | 4.35% |
|  |  | Rhizobiales | 4 | 4.35% |
|  |  | Sphingomonadales | 3 | 3.26% |
|  |  | Lactobacillales | 2 | 2.17% |
|  |  | Erysipelotrichales | 1 | 1.09% |
|  |  | Ignavibacteriales | 1 | 1.09% |
|  |  | Alteromonadales | 1 | 1.09% |
|  |  | Methanobacteriales | 1 | 1.09% |
|  |  | Bacillales | 1 | 1.09% |
|  |  | Nitrosomonadales | 1 | 1.09% |
|  | Family | unclassified | 29 | 31.52% |
|  |  | Comamonadaceae | 17 | 18.48% |
|  |  | Lachnospiraceae | 5 | 5.43% |
|  |  | Nocardiaceae | 4 | 4.35% |
|  |  | Pseudomonadaceae | 4 | 4.35% |
|  |  | Flavobacteriaceae | 3 | 3.26% |
|  |  | Burkholderiaceae | 3 | 3.26% |
|  |  | Mycobacteriaceae | 3 | 3.26% |
|  |  | Muribaculaceae | 2 | 2.17% |
|  |  | Sphingomonadaceae | 2 | 2.17% |
|  |  | Moraxellaceae | 2 | 2.17% |
|  |  | Ruminococcaceae | 2 | 2.17% |
|  |  | Bradyrhizobiaceae | 2 | 2.17% |
|  |  | SAR11 | 2 | 2.17% |
|  |  | Erythrobacteraceae | 1 | 1.09% |
|  |  | Phreatobacteraceae | 1 | 1.09% |
|  |  | Ignavibacteriaceae | 1 | 1.09% |
|  |  | Alteromonadaceae | 1 | 1.09% |
|  |  | Methanobacteriaceae | 1 | 1.09% |
|  |  | Prevotellaceae | 1 | 1.09% |
|  |  | Rikenellaceae | 1 | 1.09% |
|  |  | Bacteroidaceae | 1 | 1.09% |
|  |  | Weeksellaceae | 1 | 1.09% |
|  |  | Staphylococcaceae | 1 | 1.09% |
|  |  | Porphyromonadaceae | 1 | 1.09% |
|  |  | Lactobacillaceae | 1 | 1.09% |
| Wood | Phylum | Proteobacteria | 83 | 68.03% |
|  |  | Bacteroidetes | 15 | 12.30% |
|  |  | Acidobacteria | 7 | 5.74% |
|  |  | unclassified | 7 | 5.74% |
|  |  | Verrucomicrobia | 5 | 4.10% |
|  |  | Actinobacteria | 3 | 2.46% |
|  |  | Thaumarchaeota | 1 | 0.82% |
|  |  | Chlamydiae | 1 | 0.82% |
|  | Class | Gammaproteobacteria | 42 | 34.43% |
|  |  | Betaproteobacteria | 18 | 14.75% |
|  |  | unclassified | 16 | 13.11% |
|  |  | Alphaproteobacteria | 16 | 13.11% |
|  |  | Acidobacteria_Gp1 | 7 | 5.74% |
|  |  | Flavobacteriia | 5 | 4.10% |
|  |  | Chitinophagia | 5 | 4.10% |
|  |  | Verrucomicrobiae | 5 | 4.10% |
|  |  | Actinobacteria | 3 | 2.46% |
|  |  | Sphingobacteriia | 3 | 2.46% |
|  |  | Cytophagia | 1 | 0.82% |
|  |  | Chlamydiia | 1 | 0.82% |
|  | Order | unclassified | 36 | 29.51% |
|  |  | Burkholderiales | 17 | 13.93% |
|  |  | Pseudomonadales | 15 | 12.30% |
|  |  | Sphingomonadales | 14 | 11.48% |
|  |  | Xanthomonadales | 9 | 7.38% |
|  |  | Enterobacterales | 6 | 4.92% |
|  |  | Flavobacteriales | 5 | 4.10% |
|  |  | Chitinophagales | 5 | 4.10% |
|  |  | Verrucomicrobiales | 5 | 4.10% |
|  |  | Sphingobacteriales | 3 | 2.46% |
|  |  | Micrococcales | 2 | 1.64% |
|  |  | Rhodospirillales | 1 | 0.82% |
|  |  | Cytophagales | 1 | 0.82% |
|  |  | Rhizobiales | 1 | 0.82% |
|  |  | Nitrososphaerales | 1 | 0.82% |
|  |  | Chlamydiales | 1 | 0.82% |
|  | Family | unclassified | 51 | 41.80% |
|  |  | Moraxellaceae | 8 | 6.56% |
|  |  | Erythrobacteraceae | 8 | 6.56% |
|  |  | Rhodanobacteraceae | 7 | 5.74% |
|  |  | Burkholderiaceae | 7 | 5.74% |
|  |  | Pseudomonadaceae | 5 | 4.10% |
|  |  | Chitinophagaceae | 5 | 4.10% |
|  |  | Verrucomicrobiaceae | 5 | 4.10% |
|  |  | Sphingomonadaceae | 4 | 3.28% |
|  |  | Flavobacteriaceae | 4 | 3.28% |
|  |  | Sphingobacteriaceae | 3 | 2.46% |
|  |  | Comamonadaceae | 3 | 2.46% |
|  |  | Microbacteriaceae | 2 | 1.64% |
|  |  | Alcaligenaceae | 2 | 1.64% |
|  |  | Weeksellaceae | 1 | 0.82% |
|  |  | Acetobacteraceae | 1 | 0.82% |
|  |  | Cytophagaceae | 1 | 0.82% |
|  |  | Enterobacteriaceae | 1 | 0.82% |
|  |  | Brucellaceae | 1 | 0.82% |
|  |  | Nitrososphaeraceae | 1 | 0.82% |
|  |  | Oxalobacteraceae | 1 | 0.82% |
|  |  | Xanthomonadaceae | 1 | 0.82% |

**TABLE S6** The relative abundance of some core bacteria in nematode and wood.

| **Core bacteria** | **Nematode** | **Wood** |
| --- | --- | --- |
| *Sphingomonadales* | 2% | 5% |
| *Moraxellaceae* | 2.5% | 14% |
| *Erythrobacteraceae* | 0.3% | 1.6% |
| *Burkholderiaceae* | 3.5% | 4.4% |
| *Sphingomonadaceae* | 2.1% | 3.2% |
| *Weeksellaceae* | 0.17% | 0.43% |
| *Pseudomonadaceae* | 21.7% | 10.8% |
| *Flavobacteriaceae* | 0.5% | 0.4% |
| *Comamonadaceae* | 21.4% | 3.3% |
